# Ubiquitin B in Cervical Cancer: Critical for the Maintenance of Cancer Stem-Like Cell Characters

**DOI:** 10.1371/journal.pone.0084457

**Published:** 2013-12-18

**Authors:** Yuan Tian, Wencheng Ding, Yingying Wang, Teng Ji, Shujuan Sun, Qingqing Mo, Pingbo Chen, Yong Fang, Jia Liu, Beibei Wang, Jianfeng Zhou, Ding Ma, Peng Wu

**Affiliations:** 1 Cancer Biology Research Center, Tongji Hospital, Tongji Medical College, Huazhong University of Science and Technology, Wuhan, Hubei, P. R. of China; 2 Department of Gynecology, Third Affiliated Hospital of Zhengzhou University, Zhengzhou, Henan, P. R. of China; Wayne State University School of Medicine, United States of America

## Abstract

Cervical cancer cells exhibit an increased requirement for ubiquitin-dependent protein degradation associated with an elevated metabolic turnover rate. Ubiquitin, which is a small, highly conserved protein expressed in all eukaryotic cells, can be covalently linked to certain target proteins to mark them for degradation by the ubiquitin-proteasome system. Previous studies highlight the essential role of Ubiquitin B (UbB) and UbB-dependent proteasomal protein degradation in histone deacetylase inhibitor (HDACi) -induced tumor selectivity. We hypothesized that UbB plays a critical role in the function of cervical cancer stem cells. We measured endogenous UbB levels in mammospheres *in vitro* by real-time PCR and Western blotting. The function of UbB in cancer stem-like cells was assessed after knockdown of UbB expression in prolonged Trichostatin A-selected HeLa cells (HeLa/TSA) by measuring *in vitro* cell proliferation, cell apoptosis, invasion, and chemotherapy resistance as well as by measuring *in vivo* growth in an orthotopic model of cervical cancer. We also assessed the cancer stem cell frequency, tumorsphere formation, and *in vivo* growth of human cervical cancer xenografts after UbB silencing. We found that HeLa/TSA were resistant to chemotherapy, highly expressed the UbB gene and the stem cell markers Sox2, Oct4 and Nanog. These cells also displayed induced differentiation abilities, including enhanced migration/invasion/malignancy capabilities *in vitro* and *in vivo*. Furthermore, an elevated expression of UbB was shown in the tumor samples of chemotherapy patients. Silencing of UbB inhibited tumorsphere formation, lowered the expression of stem cell markers and decreased cervical xenograft growth. Our results demonstrate that UbB was significantly increased in prolonged Trichostatin A-selected HeLa cells and it played a key role in the maintenance of cervical cancer stem-like cells.

## Introduction

Cervical cancer is the second most common cancer among women under 65 years of age and the most frequent cause of death from gynecological cancers worldwide. A woman's risk of developing cervical cancer by 65 years of age ranges from 0.69% in developed countries to 1.38% in developing countries. In 2010, approximately 75, 000 new cases of cervical cancer were diagnosed in China [1,2]. Currently, approximately 35% of women diagnosed with cervical cancer have recurrent disease, with 90% of these found within 3 years after initial treatment [3]. Because of multi-drug resistance and also resistance to radiotherapy, conventional methods will not be effective for recurrent cases. Improved targeted therapies and chemo/radio-sensitization strategies are essential for reducing the mortality of this devastating malignancy. 

The identification of cancer stem-like cells in solid tumors opens new approaches to chemotherapy; a better understanding of the mechanisms underlying carcinogenesis and the treatment-resistant properties of cancer stem-like cells is necessary. In the last few years, accumulating evidence has suggested that the potential to initiate a tumor, including cervical carcinoma, is a rather unique feature of a small subset of stem-like cells called “cancer stem cells” (CSCs) or “tumor-initiating cells”. CSCs have the exclusive ability to self-renew, thereby expanding the pool of CSCs and allowing the tumor to differentiate into the heterogeneous non-tumorigenic cancer [4]. The CSCs hypothesis has exciting clinical implications in cervical cancer: it could explain therapy failure and disease relapse as a result of the resistance of CSCs to death stimuli. Indeed, by retaining the biological hallmarks of tissue stem cells, such as quiescence, self-renewal and multi-drug resistance, CSCs constitute an intrinsic refractory tumor cell population that is resistant to therapies developed to eradicate rapidly dividing cells. Therefore, identification and characterization of CSCs are fundamental for prognosis and treatment of cervical carcinoma [5].

In a previous study, we performed SMART to identify the key genes responsible for the tumor-selective action of Trichostatin A (TSA), one of the most extensively studied HDACis. We identified Ubiquitin B (UbB), which can covalently link to certain target proteins marking them for degradation by the ubiquitin-proteasome system (UPS), as a mediator of cancer cell apoptosis induced by TSA. Loss of UbB gene function conferred the strongest resistance to TSA treatment [6]. 

Our first finding in this study was that cells that survived TSA screening have some properties of CSCs. To confirm the characteristics of these cancer stem-like cells, we isolated sphere-forming cells (SFCs) by screening cervical cancer cell lines. Characterization of these SFCs was defined by CSC-like features, including higher tumorigenicity than parental HeLa cells; elevated expression of Sox2, Oct4 and Nanog; and multi-drug resistance. The expression of UbB in these stem-like cells was examined in further research. We found that the maintenance of cancer stem-like characteristics correlated with UbB expression, and silencing UbB expression could reverse the cancer stem cell characteristics.

## Materials and Methods

### Ethics Statement

Twenty-two patients who diagnosed cervical squamous cell carcinoma and underwent primary surgery directly or after received regular Cisplatin-based chemotherapy were chosen to participate in this study and all patients provided written informed consent. The postoperative tissue samples were collected by the Department of Clinical Pathology, Tongji Hospital, Tongji Medical College, Huazhong University of Science and Technology (HUST). Information on histopathologic diagnosis was reviewed by at least two specialists in gynecologic pathology. This study was reviewed and approved by the Ethics Committee of Tongji Hospital, Tongji Medical College, HUST.

### Xenograft tumor study

These experiments were performed using 6- to 8-week-old female nonobese diabetic/severe combined immuno-deficient (NOD/SCID) mice purchased from HFK (Beijing) and housed at the Experimental Animal Center, Tongji Medical College, HUST. Mice were selected randomly for each group; a total of six mice per group were used and housed at three per cage. HeLa and HeLa/TSA cells were trypsinized, counted, and re-suspended in 100 µl of Dulbecco’s PBS and then injected subcutaneously. The growth rates of xenografts were measured every three days, and the volume was calculated by using of the formula: L×W^2^×0.5. The photographs of xenografts were taken after mice were given a lethal overdose of sodium pentobarbital anesthesia. All experimental protocols were approved by the Institutional Animal Care and Use Committee of HUST, and the study was carried out in strict accordance with the ARRIVE (Animal Research: Reporting of *In Vivo* Experiments) guidelines [7]{Kilkenny, 2012 #29}.

### Cell culture and transfection

HeLa cells were obtained from ATCC and maintained in our lab. HeLa/TSA cells were established by treating HeLa cells with 1 μM Trichostatin A (TSA, sigma) for 24 hours and then maintaining the cells in 200 nM TSA for another 7 to 10 days. The surviving cells were allowed to recover for another 4-7 days. Then, they were collected and allowed to proliferate.

The siRNAs (Invitrogen) were transfected using Lipofectamine 2000 (Invitrogen) according to manufacturer’s instructions. An shRNA lentivirus was utilized to infect cells following the manufacturer’s protocol.

### UV exposure of the cells

For UV irradiation, cells were seeded at a density of 2×10^5^/ml and grown until attachment was achieved and an even monolayer was formed. Then, the cells were washed twice with prewarmed PBS and exposed to UV while in PBS. UV light was generated from a 15-W UVB lamp (UVP), which emits most of the energy within the UVB range of 280 - 370 nm, with an emission peak at 310 nm. The intensity of UVB was standardized by a UVB meter and set at 200J/m^2^. Following irradiation, fresh medium was added.

### Transwell migration assay

HeLa and HeLa/TSA cells were seeded into a transwell chamber for 48 hours. The cells that migrated through the membrane were fixed and stained with 0.1% crystal violet and then examined under a light microscope.

### Identification of SP cells

HeLa and HeLa/TSA cells were trypsinized and incubated with 5 μg/mL Hoechst 33342 dyes (Roche) at 37°C for 90 min; the reaction was terminated by incubation in ice water for 10 min. The dyed cell samples were analyzed by a FACSCalibur flow cytometer (BD) using a 355-nm UV excitation; the fluorescence emission was collected using a 450-nm band-pass filter for Hoechst blue and a 670-nm band-pass filter for Hoechst red. Data acquisition and analysis were performed with CellQuest Pro software (BD).

### Colony formation, mammosphere formation and limiting dilution assays

HeLa and HeLa/TSA cells were counted and plated at the same density into a 6-well plate for 7 days. The cells were washed twice with PBS and fixed with 4% paraformaldehyde for 10 minutes. Then, the cells were washed with distilled water for 5 minutes twice and incubated with a 0.1% crystal violet staining solution for 10 minutes. Finally, the cells were washed with distilled water for 5 minutes twice or until the excess dye was completely removed.

Mammosphere formation and limiting dilution assays were performed as described previously by Calcagno AM [8]. Generally, we performed these experiments in HeLa and HeLa/TSA cells to assess mammosphere formation in ultra-low attachment culture wells (Costar) in serum-free DMEM/F12 medium supplemented with recombinant human epidermal growth factor (EGF, 10 ng/mL, Peprotech); recombinant human fibroblast growth factor-basic (bFGF, 10 ng/mL, Peprotech); insulin (50 μg/mL, Sigma); B27 (100 units/mL, Invitrogen), penicillin (100 units/mL, Invitrogen) and streptomycin (100 μg/mL, Invitrogen). Mammospheres were identified as described [9] every 3 days according to colony proliferation rates. The limiting dilution assay was performed by plating various numbers of cells (from 500 cells to only one cell) per well into three 96-well ultra-low attachment plates. Spheroids were counted at 14 days or later, depending on the growth rates of the spheroids. The experiments were conducted in triplicate, and the calculated averages are presented. 

### RNA isolation, reverse transcription and quantitative RT-PCR analysis

Total RNA was isolated using the Qiagen RNeasy kit according to the manufacturer’s instructions. RNA quantitation was determined using a NanoDrop micro-volume spectrophotometer (Thermo Fisher), and the mRNA integrity was verified by agarose gel electrophoresis. Reverse transcription-polymerase chain reaction (RT-PCR) was then performed using 2 μg of total RNA. Quantitative RT-PCR was performed in a CFX96 Touch^TM^ Real-Time PCR Detection System (Bio-Rad) using the following thermocycler program for all genes: 5 min of pre-incubation at 95°C followed by 40 cycles of 15 s at 95°C, 15 s at 60°C, and 30 s at 72°C. The primers of all target genes and the reference gene are listed in Table S1. The data collection, including the fold change in gene expression, was determined from the same amount of total RNA. 

### Western blotting analysis

 Total cell proteins were extracted and resolved by 10% SDS-PAGE, transferred to PVDF polyvinylidene difluoride (PVDF) membranes (350mA for 1 h), and probed with antibodies against UbB, UbC, UbA52, UbA80, pSTAT3, Vimentin, pAKT and β-actin (ProteinTech Group), Mdr-1 and Oct4 (Santa Cruz), Sox2 and Nanog (Millipore). Proteins were visualized using Horseradish peroxidase conjugated secondary antibodies with SuperSignal West Pico Chemiluminescent Substrate (Pierce) and photographed in ChemiDoc™ XRS+ System with Image Lab™ Software (Bio-Rad).

### Immunofluorescence of mammospheres

Mammospheres were collected after approximately 14 days, depending on size and growth rates, and then washed with PBS twice and centrifuged at 300 g. The mammospheres were re-suspended with Optimal cutting temperature compound (O.C.T., Sakura) and placed in liquid nitrogen. Frozen spheroids were then sliced into 5-μm-thick sections. Immunofluorescence of these frozen sections was performed according to the primary antibody manufacturer’s protocol. Fluorescence imaging was performed using a confocal laser scanning microscope.

### Flow cytometry

HeLa and HeLa/TSA cells were treated for 48 hours with 1μM TSA, 30 μM DDP (Sigma, P4393), or 75 nM Paclitaxol or were irradiated with UVB. Then, the cells were trypsinized and incubated with Annexin V-FITC and PI according to the manufacturer’s protocol. The apoptosis percentage was determined using BD FACSCalibur flow cytometer and CellQuest Pro software (BD).

### Statistical Methods

Statistical significance was determined by the unpaired Student’s t test using GraphPad Prism software (Version 5.01), except for the comparison of UbB expression in clinical cervical cancer tissue samples, which was analyzed via the two-way ANOVA test. Significance for all tests was set at *p*<0.05.

## Results

### Prolonged HDACi-selected HeLa/TSA cells were resistant to chemotherapy

**Figure 1:**
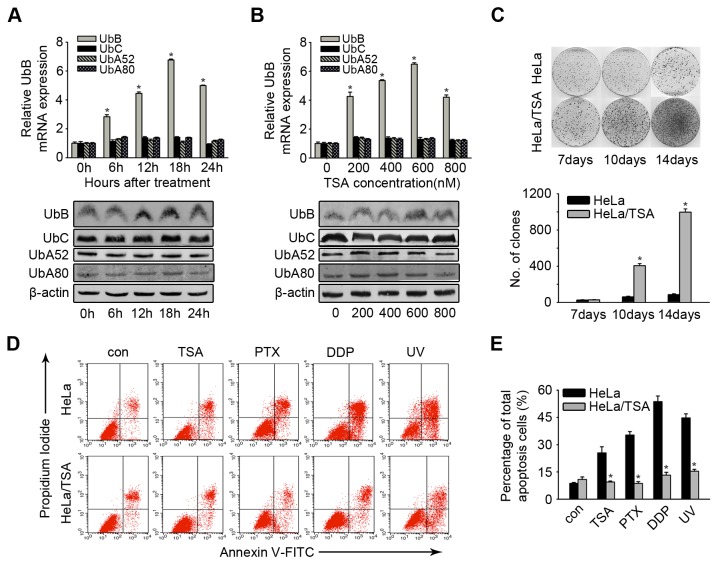
The increasing effect of colony formation and drug-resistance in HeLa/TSA. **A**, HeLa cells were treated with 500 nM TSA for the indicated times and subjected to analysis the mRNA level of UbB, UBC, UbA52 and UbA80 by real-time PCR and the corresponding protein level by western blotting. **B**, HeLa cells were incubated with TSA for 24 hours at a dose ranging from 200 to 800 nM and subjected to analysis the mRNA level of UbB, UbC, UbA52 and UbA80 by real-time PCR and the corresponding protein level by western blotting. **C**, Upper panel: representative dishes of the colony forming assay at day 7, 10 and 14. Lower panel: numbers of colony formed in HeLa and HeLa/TSA at day 7, 10 and 14. The columns represent the average of three separate experiments; error bars, SD; *, *p*<0.05. **D**, HeLa/TSA cells were treated with TSA (1 μM), DDP (30 μM), and PTX (75 nM) for 48 h or UV, the apoptosis cells were quantified by Annexin V/PI staining and the flow cytometry analysis. The representative examples of the flow cytometry results were shown. **E**, Cells were treated as described in **D**, the average percentages of apoptosis cells were reported in the graphs. Values, mean percentages; error bars, SD; *, *p* < 0.05 (n = 3 replications).

Incubation of HeLa cells with TSA (500 nM) at different time points (6, 12, 18 and 24 hours) induced a time-dependent increase in UbB mRNA and Ubiquitin protein levels (Figure 1A), while the expressions of the other three ubiquitin-coding genes (UbA52, UbA80 and UbC) were not affected. Similarly, incubation with different concentrations (200, 400, 600 and 800 nM) of TSA for 24 hours also resulted in an increase of UbB gene expression, while no change of expressions of the other three ubiquitin-coding genes were observed. TSA administration, within a certain concentration range, for 24 hours led to a specific concentration-dependent stimulation of UbB mRNA and protein expression (Figure 1B).

Until the majority of HeLa cells underwent apoptosis after treatment with TSA (1 μM for 24 h and maintained with 200 nM for 7 to 10 days), the surviving cells (HeLa/TSA) were seeded to form clones. After another 10 to 14 days of growth, crystal violet staining showed that HeLa/TSA cells had a significant proliferative ability compared with the corresponding control cells. The number of HeLa/TSA colonies was approximately 16-fold more than that of the control on the 14th day (*p*<0.01) (Figure 1C). 

To further examine the sensitivity of HeLa/TSA cells to TSA, we treated HeLa/TSA cells with TSA and other common chemotherapy agents. As shown in the FACS results, HeLa/TSA cells demonstrated a notable resistance to not only TSA (mean 9.48% vs. 25.53%, *p*=0.014) but also Cisplatin (DDP) (mean 13.25% vs. 53.76%, *p*<0.01), Paclitaxol (PTX) (mean 8.69% vs. 35.36%, *p*<0.01), and even ultraviolet light (UV) (mean 15.38% vs. 44.8%, *p*<0.01) (Figure 1D, E). These results suggest that HeLa/TSA cells may contain an enriched subset of cells resistant to common chemotherapy agents, even UV.

### HeLa/TSA cells exhibited an up-regulation of UbB expression and increased mammosphere formation

**Figure 2:**
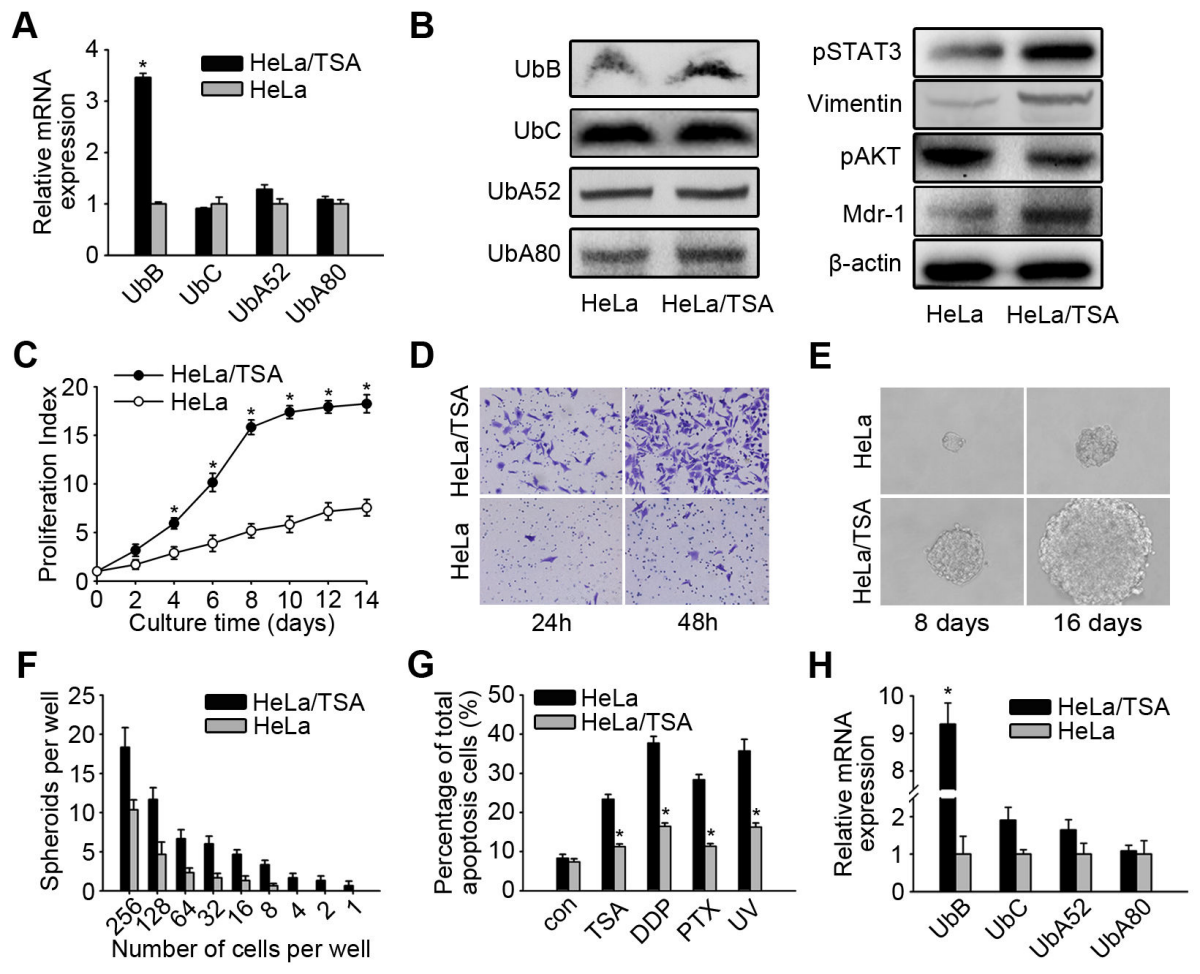
HeLa/TSA was up-expressed UbB and exhibited higher ability of the mammosphere formation. **A**, HeLa/TSA cells were analyzed for UbB, UbC, UbA52 and UbA80 mRNA levels. The columns represent the average of three separate experiments; error bars, SD; *, *p*<0.05. **B**, HeLa/TSA cells were analyzed for the protein levels of Ub proteins, pSTAT3, Vimentin, pAKT and Mdr-1, β-actin was used as a loading control. **C**, Quantitative analysis of the proliferation index determined by the CCK-8 assay for the indicated times. The results shown are averaged through three separate experiments; error bars, SD. **D**, Representative examples of tranwell assay were photographed at 200× magnifications. **E**, Themorphologic appearance of a representative mammosphere for the indicated times was photograghed at 200×magnifications. **F**, Spheroid formation assays were performed by limiting dilution with 256 cells to one cell per well of a 96-well Ultra-Low Adherent plate. This experiment was performed for triple times with six wells per well dilution. The number of colonies was counted at day 14. **G**, Mammosphere cells of HeLa and HeLa/TSA were re-cultured in an ordinary adherent plate for 24 h, and then treated with TSA, DDP, and PTX for 48 hours or UV for 5 min. The apoptosis cells were quantified using Annexin V/PI staining and the flow cytometry analysis. The values are average and SD (error bars) of 3 separate experiments, *, *p*<0.05. **H**, HeLa/TSA mammosphere cells were analyzed for UbB, UbC, UbA52 and UbA80 mRNA levels. GAPDH was used as a reference control.

To further investigate the resistance of HeLa/TSA cells to chemotherapy and UV light was related to the expression of UbB, the mRNA expression in HeLa/TSA cells was evaluated. We observed a significant increase of UbB in HeLa/TSA cells, while no apparent change of the other three ubiquitin genes (Figure 2A). Similarly, we also found a significant increase in protein level of UbB, not the other three ubiquitin-coding genes. Moreover, we also found up-regulation of pSTAT3, Vimentin and Mdr-1 along with down-regulation of pAKT in HeLa/TSA cells when compared with the parental HeLa cells (Figure 2B). These data indicated that HeLa/TSA cells were chemo-resistant and it may be related to the high expression of UbB. Moreover, the majority of HeLa/TSA cells were in an undifferentiated state and undergoing epithelial-mesenchymal transition (EMT). The findings reported here are consistent with our previous results, which indicated that UbB is an essential mediator of TSA-induced tumor-selective killing [6].

We further examined the biological characteristics of HeLa/TSA cells and observed that the proliferation of HeLa/TSA cells was significantly higher than in HeLa control cells, especially in long-term culture (Figure 2C). In addition, the rate of migration in HeLa/TSA cells was significantly increased in the Transwell Migration Assay (Figure 2D). These results suggested that HeLa/TSA cells exhibited a high malignant potential.

Previously reported studies have indicated that a prolonged continuous selection of cells for drug resistance enriches cell populations with cancer stem-like cell characteristics [8]. The data reported above demonstrated that HeLa/TSA cells showed a high malignant potential, which may have emerged under the selective pressure of chemotherapy. We performed experiments to test whether HeLa/TSA cells were enriched with cancer stem-like cells. HeLa/TSA cells were cultured in a non-adherent state to form mammospheres. We found that HeLa/TSA cells had a significantly higher mammosphere formation potential. The HeLa/TSA mammospheres were larger and grew more rapidly than the HeLa mammospheres, which were small cell clusters that adhered to the dish and were difficult to score in the limiting dilution assay (Figure 2E). Although HeLa cells had a tendency to form mammospheres, they were unable to be scored after seeding fewer than eight cells, whereas only one HeLa/TSA cell had a probability of 66.7% to form a mammosphere (Figure 2F). To ascertain whether spheroids were identical to adherently cultured cells, we trypsinized spheroids and recultured them in an adherent state; these re-adherent HeLa/TSA cells were still resistant to TSA, DDP, PTX and UV, in contrast to the results for re-adherent HeLa cells, which were not resistant (Figure 2G). Furthermore, UbB gene expression in HeLa/TSA mammospheres was still significantly higher than in the control HeLa mammosphere cells (Figure 2H).

The above data indicated that HeLa/TSA cells exhibited an up-regulation of UbB, pSTAT3, Vimentin, whereas pAKT was down-regulated. Moreover, these cells were undifferentiated and in a state of quiescence and exhibited a higher mammosphere-forming ability in a non-adherent situation. These characteristics highlighted that HeLa/TSA cells may contain cancer stem-like cells, and this phenomenon could be related to the up-regulation of UbB.

### HeLa/TSA cells have cancer stem-like properties

**Figure 3:**
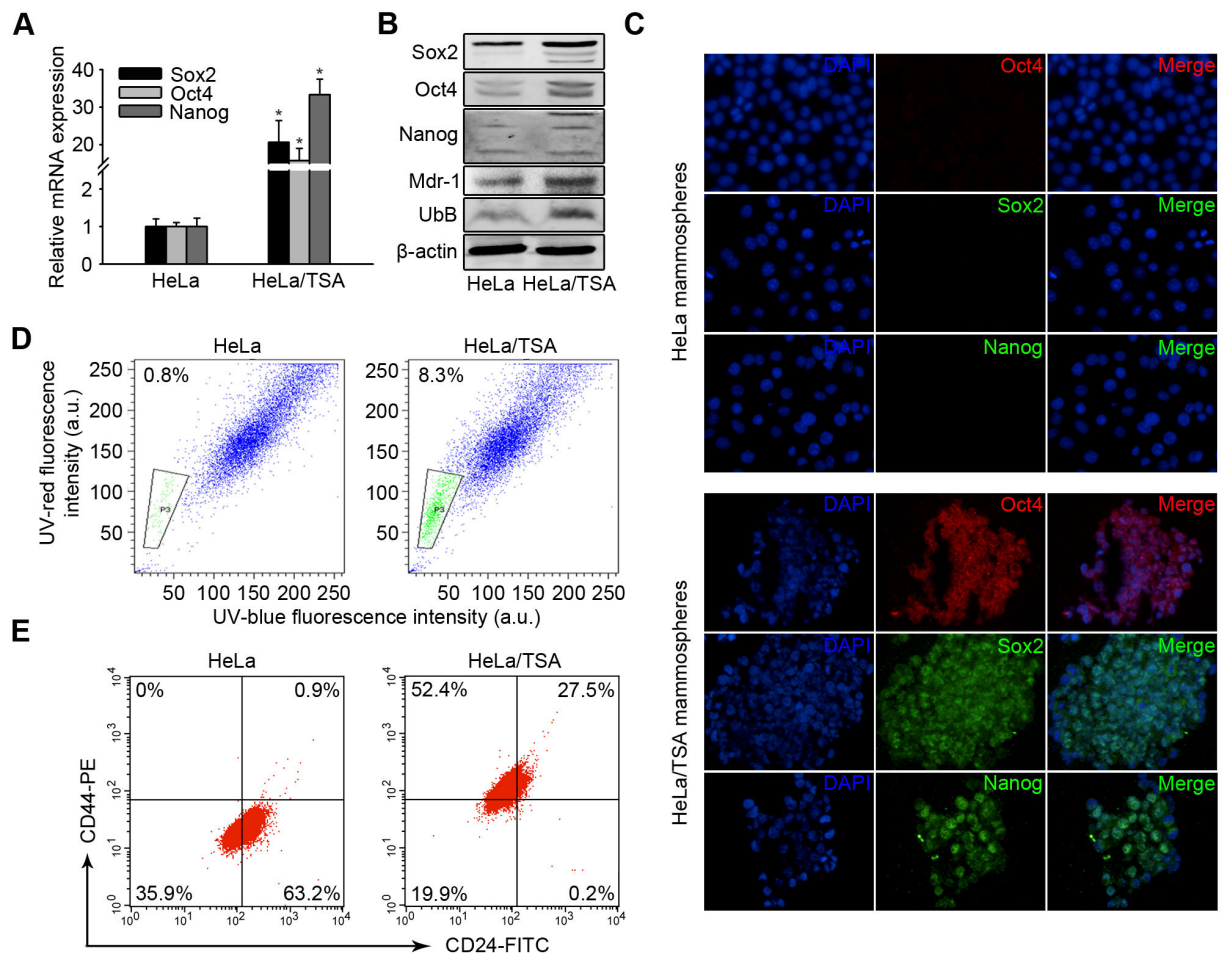
HeLa/TSA cells were enriched of cancer stem-like cells. **A**, HeLa/TSA mammosphere cells were analyzed for the Sox2, Oct4 and Nanog mRNA levels. The columns represent the average of three separate experiments; error bars, SD. **B**, The quantitative analysis of Sox2, Oct4, Nanog, Mdr-1 and UbB protein levels by western blotting. **C**, HeLa/TSA mammosphere cells were analyzed by immunofluorescence for Sox2, Oct4 and Nanog. The representative photographs were taken using a fluorescence microscope (original magnification, ×200). The nuclei were presented as detected using *DAPI* (4′, 6-Diamidino-2-phenylindole) staining. **D**, The analysis of the side-population cell fraction in HeLa and HeLa/TSA cell cultures. Left panel: HeLa; right panel: HeLa/TSA. E, The analysis of CD44 and CD24 expression of HeLa and HeLa/TSA cells. Left panel: HeLa; right panel: HeLa/TSA.

To further examine whether HeLa/TSA cells were enriched with cancer stem-like cells, we performed real-time PCR to detect the gene expression of stem cell biomarkers. We found that Sox2, Oct4, and Nanog were significantly up-regulated in HeLa/TSA cells compared with HeLa mammospheres (Figure 3A). Western blotting confirmed that HeLa/TSA mammospheres highly expressed the stem cell biomarkers. In addition, we also found Mdr-1 and UbB were high expressed in HeLa/TSA cells (Figure 3B). Similarly, immunofluorescence assay results showed that almost all the HeLa/TSA cells possessed the primary features of stem-like cells, namely, the expression of those stem cell biomarkers (Figure 3C).

It has been reported that the ABC transporter-mediated efflux employed by the Hoechst 33342 dye to isolate dye-excluding side populations (SPs) is enriched in cells with cancer stem-like properties in a variety of tumors [10]. Using differential Hoechst 33342 staining to identify SPs in adherent cells, we observed a significant increase of SP-positive cells in HeLa/TSA cells compared with parental HeLa cells (mean 8.3% vs. 0.8%, *p*<0.01, paired student’s t test) (Figure 3D). Previous studies showed that breast CSCs from mammosphere cells exhibited surface markers of CD44^+^CD24^-^[9,11]. As HeLa/TSA exhibited a high formation rate of spheroids, we examined CD44 and CD24 expression in HeLa/TSA cells. The results showed that 52.4% of HeLa/TSA was CD44^+^CD24^-^, which was similar to the previous reported in breast CSCs (Figure 3E).

These data showed that prolonged drug selection of HeLa/TSA cells induced a high expression of stem cell biomarkers and an enrichment of CD44^+^CD24^-^ cells. Therefore, HeLa/TSA cells may be enriched of the cells which have cancer stem-like properties. 

### UbB siRNA could down-regulate SP and spheroid formation

**Figure 4:**
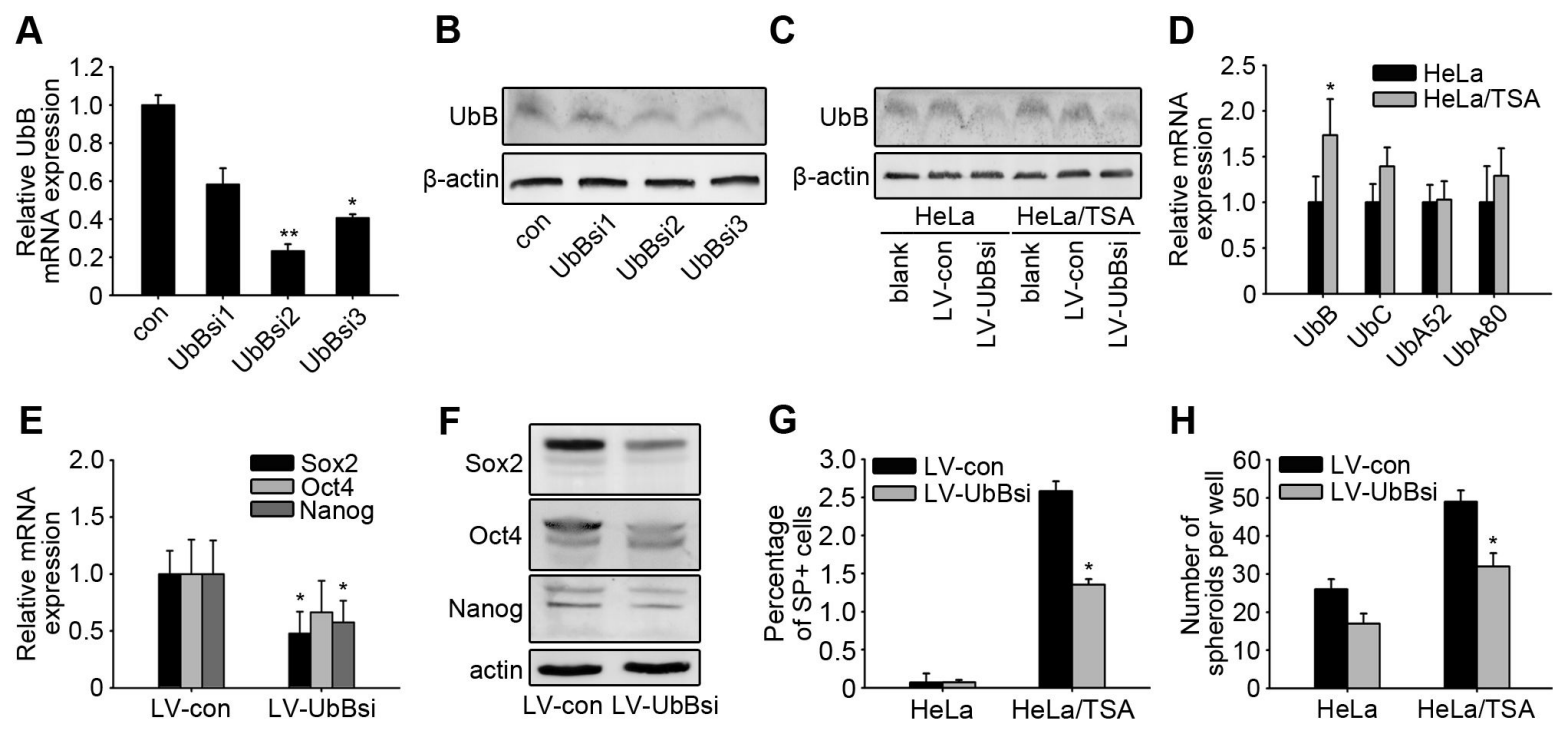
UbB siRNA could down-regulate the SP and spheroids formation. **A**, HeLa cells were transfected with 10 nM UbB-targeting siRNA, or stealth RNAi control, for 72 hours. Real-time PCR analysis of relative UbB mRNA expression was proceeded. GAPDH was used as a reference control. **B**, The quantitative analysis of the UbB protein level by western blotting. Cells were treated as described in A. Beta-actin was used as a reference control. **C**, HeLa and HeLa/TSA mammosphere cells were infected with LV-UbBsi, which was cloned with UbBsi2, for 72 hours and subjected to analysis by western blotting for UbB protein level. **D**, LV-UbBsi-infected mammosphere cells were analyzed for the UbB, UbC, UbA52 and UbA80 mRNA levels. GAPDH was used as a reference control. The columns represent the average of three separate experiments; error bars, SD. **E**, LV-UbBsi-infected HeLa/TSA mammosphere cells were analyzed for the Sox2, Oct4 and Nanog mRNA levels. GAPDH was used as a reference control. The columns represent the average of three separate experiments; error bars, SD. **F**. LV-UbBsi-infected HeLa/TSA mammosphere cells were analyzed for the Sox2, Oct4 and Nanog protein levels. Beta-actin was used as a reference control. **G**, The analysis of the side-population cell fraction in LV-UbBsi-infected HeLa and HeLa/TSA cell cultures. **H**, The spheroid formation assays were performed on LV-UbBsi-infected cells.

Resistance of cancer stem cells to chemotherapeutic drugs has been previously described in a variety of tumor types, including ovarian cancer [10]. To confirm that the malignant phenotype and high SP cell ratio of HeLa/TSA cells were related to the high expression of the UbB gene, we utilized siRNA to knockdown UbB in cells. Following transfection of HeLa cells with 3 different siRNAs that target the CDS of the UbB gene, we elected to use UbBsi2 because it showed a 77% knockdown of the UbB gene according to real-time PCR and western blotting results (Figure 4A, B). To effectively transfect siRNAs into spheroids, we introduced UbBsi2 into a lentivirus vector (LV-UbBsi). After knockdown of the UbB gene in both HeLa and HeLa/TSA spheroids using LV-UbBsi (Figure 4C), real-time PCR results showed that although UbB gene expression of HeLa/TSA-LV-UbBsi spheroids was still significantly higher than that of HeLa-LV-UbBsi spheroids (*p*<0.01) (Figure 4D), the difference between these groups was only 1.74-fold, which was much lower than the 9.25-fold difference between the non-silenced HeLa/TSA and control HeLa spheroids (Figure 2H). Meanwhile, the expression of Sox2, Oct4 and Nanog in HeLa/TSA cells was reduced significantly after LV-UbBsi infection (Figure 4E, F), and the SP^+^ ratio of HeLa/TSA-LV-UbBsi cells was significantly reduced from 2.58% to 1.25% (*p*<0.01) (Figure 4G). After re-culturing the UbB-silent spheroids in suspension, we observed that the number of spheroids formed in HeLa/TSA-LV-UbBsi cells was reduced from a mean of 49 to 32 (*p*=0.04) (Figure 4H). These data showed that silencing UbB visibly reduced the tumor stem-like properties of HeLa/TSA cells suggesting that cancer stem-like properties of HeLa/TSA cells may be related to the high expression of UbB.

### Fewer HeLa/TSA cells could form xenografts *in vivo*

**Figure 5:**
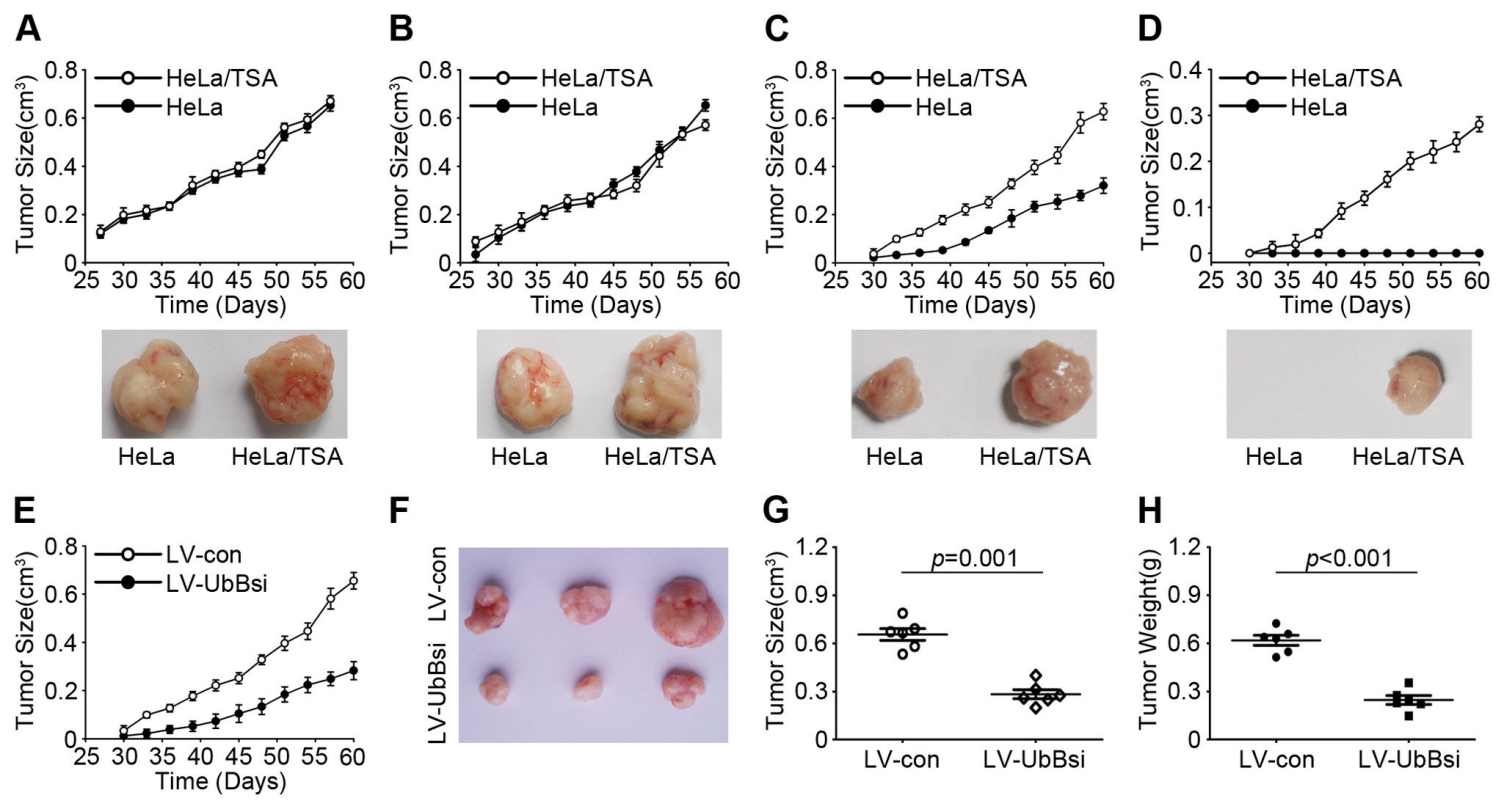
Fewer HeLa/TSA could form xenografts *in* *vivo*. HeLa and HeLa/TSA cells were injected subcutaneously into NOD/SCID mice with four different cell densities. **A**-**E**, The tumor volume is plotted as a function of time. **A**, 5000 cells; **B**, 1000 cells; **C**, 500 cells; **D**, 100 cells; **E**, 500 LV-con or LV-UbBsi-infected HeLa/TSA cells. **F**, The photographs of subcutaneously formed LV-con or LV-UbBsi-infected HeLa/TSA tumors are shown. **G**, The tumor size decreased with respect to the UbB inhibition following the LV-UbBsi infection in HeLa/TSA xenografts. The horizontal lines represent the average size; error bar, SD; *p*=0.001, two-way ANOVA test; 6 mice/group. **H**, The tumor weights of xenografts described in **G**. The horizontal lines represent the average weight; error bar, SD; *p*<0.001, two-way ANOVA test; 6 mice/group.

To further validate the stem-like potential of HeLa/TSA cells, we performed *in vivo* experiments by subcutaneously injecting 100 to 5000 cells into NOD/SCID mice. We could not detect any difference in tumorigenicity between HeLa/TSA and HeLa cells when injecting more than 1,000 cells (Figure 5A, B). However, when less than 500 cells were implanted, HeLa/TSA cells exhibited a potentially more tumorigenic state (Figure 5C, D). In addition to the differences in average tumor growth, HeLa/TSA cells initiated tumors more frequently (6/6 injections) than parent HeLa (3/6 injections) cells (*p*=0.04) in response to an injection of 500 cells (Table 1). Furthermore, HeLa/TSA cells initiated tumors in 6/6 injections, while HeLa cells did not initiate tumors (0/6 injections) when 100 cells were implanted. The greater ability for tumor growth *in vivo* was consistent with malignant growth in adherent cell culture. After knockdown of UbB by LV-UbBsi, although HeLa/TSA-LV-UbBsi could form xenografts at the same frequency compared to HeLa/TSA-LV-con when injected with 500 infected cells (Figure 5E), the size and weight of the xenografts were significantly reduced (Figure 5F, G, H). These results confirmed that HeLa/TSA cells exhibited a high tumorigenic potential that could be reduced by silencing UbB.

**Table 1:** Evaluation of tumorigenicity of HeLa and HeLa/TSA cells.

Cell line	No. of cells injected	No. of tumors /No. of mice	Maximum tumor volume (mm^3^)	Maximum tumor weight (g)	Day of tumor harvest
HeLa	5000	6/6	675	0.48	51
	1000	5/6	628	0.41	54
	500	4/6	668	0.46	57
	100	0/6	☆	0	☆
HeLa/TSA	5000	6/6	852	0.59	51
	1000	6/6	573	0.39	54
	500	6/6	810	0.53	57
	100	6/6	570	0.39	60

☆ No palpable tumors were present after 60 days

### Increased expression of UbB in cervical cancer tissues from patients with chemotherapy compared with non-chemotherapy

**Figure 6:**
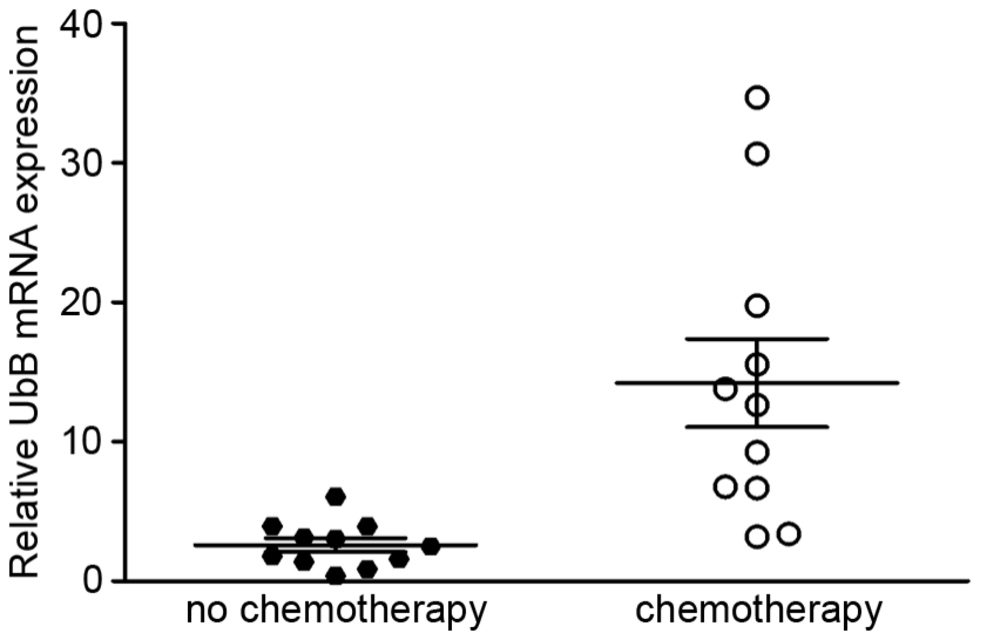
Relative expression of UbB in cervical cancer tissue samples. The expression of UbB in cervical cancer tissue samples with collected either initial presentation or after platinum-based chemotherapy were analysed. The UbB expression level was significantly higher in the chemotherapeutic samples than that in non-chemotherapeutic samples (*p*=0.0059, two-way ANOVA test; n=22).

We evaluated the relationship between UbB expression and chemotherapy by measuring the relative UbB expression of cancer tissue for comparison with the adjacent tissues. We found that the relative expression of UbB in cervical cancer without chemotherapy ranged from 0.85 to 6.05, compared to adjacent tissues, while cervical cancer tissue following chemotherapy exhibited a UbB expression from 3.19 to 34.69 (Figure 6) (Table 2). The expression of UbB shows a significant increase in cervical cancer following chemotherapy compared with non- chemotherapy (*p*=0.0059, two-way ANOVA test). These data indicate that the surviving cancer cells after chemotherapy highly express UbB, which may be the main cause of chemotherapy resistance.

**Table 2:** Clinical characteristics and UbB gene expression in cervical cancer ascites.

Sample number	Age	Grade	Matastasis	Histology	chemotherapy	UbB gene expression*	SD
1	34	Ib1	N	SCC	N	2.504	0.016
2	40	Ib1	N	SCC	N	3.916	0.042
4	41	Ib2	N	SCC	N	1.592	0.163
8	41	Ib2	N	SCC	N	1.393	0.245
9	28	Ib2	N	SCC	N	6.047	0.015
12	38	IIa	Y	SCC	N	3.114	0.101
15	47	Ib2	N	SCC	N	0.855	0.083
16	45	Ib1	N	SCC	N	1.792	0.163
17	39	Ib2	N	SCC	N	0.393	0.245
18	47	Ib1	N	SCC	N	3.047	0.015
22	39	Ib2	N	SCC	N	3.941	0.120
3	39	Ib2	N	SCC	Y	6.786	0.249
5	47	Ib1	N	SCC	Y	3.187	0.084
6	39	Ib1	N	SCC	Y	6.671	0.129
7	47	Ib2	N	SCC	Y	30.666	0.123
10	39	Ib2	N	SCC	Y	12.639	0.142
11	43	IIa	Y	SCC	Y	34.692	0.368
13	42	IIb	Y	SCC	Y	19.754	0.124
14	45	Ib1	N	SCC	Y	15.559	0.048
19	41	Ib1	N	SCC	Y	13.786	0.295
20	39	Ib2	N	SCC	Y	9.249	0.064
21	40	Ib1	N	SCC	Y	3.388	0.086

^*^ Relative UbB mRNA expression of cervical cancer tissue samples contrast to the adjacent tissues. SCC: squamous cell carcinoma. Y: Yes. N: No. SD: standard deviation.

## Discussion

Histone deacetylase inhibitors (HDACis) play important roles in epigenetic and non-epigenetic regulation, including death, apoptosis, and cell cycle arrest in cancer cells, while normal cells are relatively more resistant to HDACi-induced cell death [12]. Two FDA-approved HDACis, Vorinostat and depsipeptide, have great clinical benefits and minimal drug-related adverse effects (AEs) when used to treat hematological malignancies, such as cutaneous T cell lymphoma (CTCL). However, the clinical outcomes of HDACis, including vorinostat and depsipeptide, are disappointing when used as a single agent to treat solid tumors [13]. As shown in our previous results, one of most studied HDACis, TSA, could selectively induce apoptosis of tumor cells [6]. However, little attention has been paid to the cells that survive TSA treatment. We collected and amplified the surviving cells (HeLa/TSA) and found that they have some important features that may contribute to chemoresistance.

It has been reported that MCF-7/ADR, invasive breast cancer cells that were selected with doxorubicin for a prolonged amount of time, formed mammospheres and were tumorigenic in mice. In contrast to parental MCF-7 cells, more than 30% of MCF-7/ADR cells had a CD44^+^/CD24^-^phenotype, including self-renewal, differentiation, and over-expression of various multidrug resistance–linked genes (ABCB1, CCNE1 and MMP9) [8]. Our results showed that more than 50% of HeLa/TSA cells had a CD44^+^/CD24^-^phenotype, and they were high-expressed UbB and Mdr-1 gene. Moreover, HeLa/TSA cells were not only resistant to TSA but also to DDP, PTX, and even UV. These cells exhibited a high proliferative and invasive potential. They massively proliferated in non-adherent culture conditions and in *in vivo* mouse models, and the markers of cancer stem-like cells, Sox2, Oct4 and Nanog, were highly expressed. Although we have not found any proof of self-renewal and differentiation in HeLa/TSA cells, these data demonstrate that HeLa/TSA cells possess some characteristics of cancer stem-like cells.

CSCs have been reported to be one of the reasons for relapse or recurrence of various cancers because they are resistant to common chemotherapy agents or radiotherapy [14]. CSCs have been found in many malignant cancers, such as ALL, breast and colon cancers [4]. In ovarian cancer, SP cells can be detected. They have been reported to have characteristics of CSCs and could be SP-enriched in ovarian cancer, thus indicating that CSCs may contribute to the drug resistance of ovarian cancer [10]. Recently, SPs and CSCs have been noted in a few reports regarding cervical cancer. By applying standard techniques to investigate CSCs, we detected SP cells and a high expression of cancer stem-like cell markers in HeLa/TSA cells, which indicated that prolonged selection of cervical cancer cells by chemotherapy agents, may lead to the accumulation of SPs and CSCs.

Ubiquitin is a small, highly conserved protein expressed in all eukaryotic cells that can be covalently linked to certain target proteins, thereby marking them for degradation by the ubiquitin-proteasome system. The abundance of cellular Ubiquitin is a dynamic balance ultimately maintained by the *de novo* synthesis of ubiquitin from ubiquitin gene transcripts. Human ubiquitin is encoded by a family of multiple genes, including UbA52, UbA80, UbB, and UbC, of which both UbB and UbC are inducible by various cell stressors [15–18]. In our study, we first showed that cells that expressed UbB possessed a higher potential for colony formation *in vitro* and tumorigenicity *in vivo*. Most notably, we showed that UbB is important for maintaining the properties of CSCs and cancer initiation. The percentage of SP-positive cells and the number of mammospheres per unit cells of HeLa/TSA were significantly reduced after interference by LV-UbBsi. Additionally, we found that cancer stem-like cell markers, such as Sox2, Oct4 and Nanog, were down-regulated when UbB was knocked down, suggesting a crosstalk between UbB signaling and the expression of cancer stem-like cell markers. These data, for the first time, experimentally implicate UbB as an important regulator in maintaining cancer stem-like characteristics and cervical cancer initiation. Further study is underway to delineate the role of UbB in the complex regulatory transcriptional programs that control the renewal and maintenance of the cancer stem cell state. 

## Supporting Information

Table S1 **Quantitative RT-PCR primers used in this study.**
(XLS)
