# Correction: Ubiquitin B in Cervical Cancer: Critical for the Maintenance of Cancer Stem-Like Cell Characters

**DOI:** 10.1371/journal.pone.0152813

**Published:** 2016-03-28

**Authors:** Yuan Tian, Wencheng Ding, Yingying Wang, Teng Ji, Shujuan Sun, Qingqing Mo, Pingbo Chen, Yong Fang, Jia Liu, Beibei Wang, Jianfeng Zhou, Ding Ma, Peng Wu

The authors would like to correct Fig 1, as errors were introduced in the preparation of this figure for publication. In Fig 1C, the panels for HeLa at 7 days and at 10 days appear to be identical. The authors have provided a corrected version of Fig 1 here.

**Fig 1:**
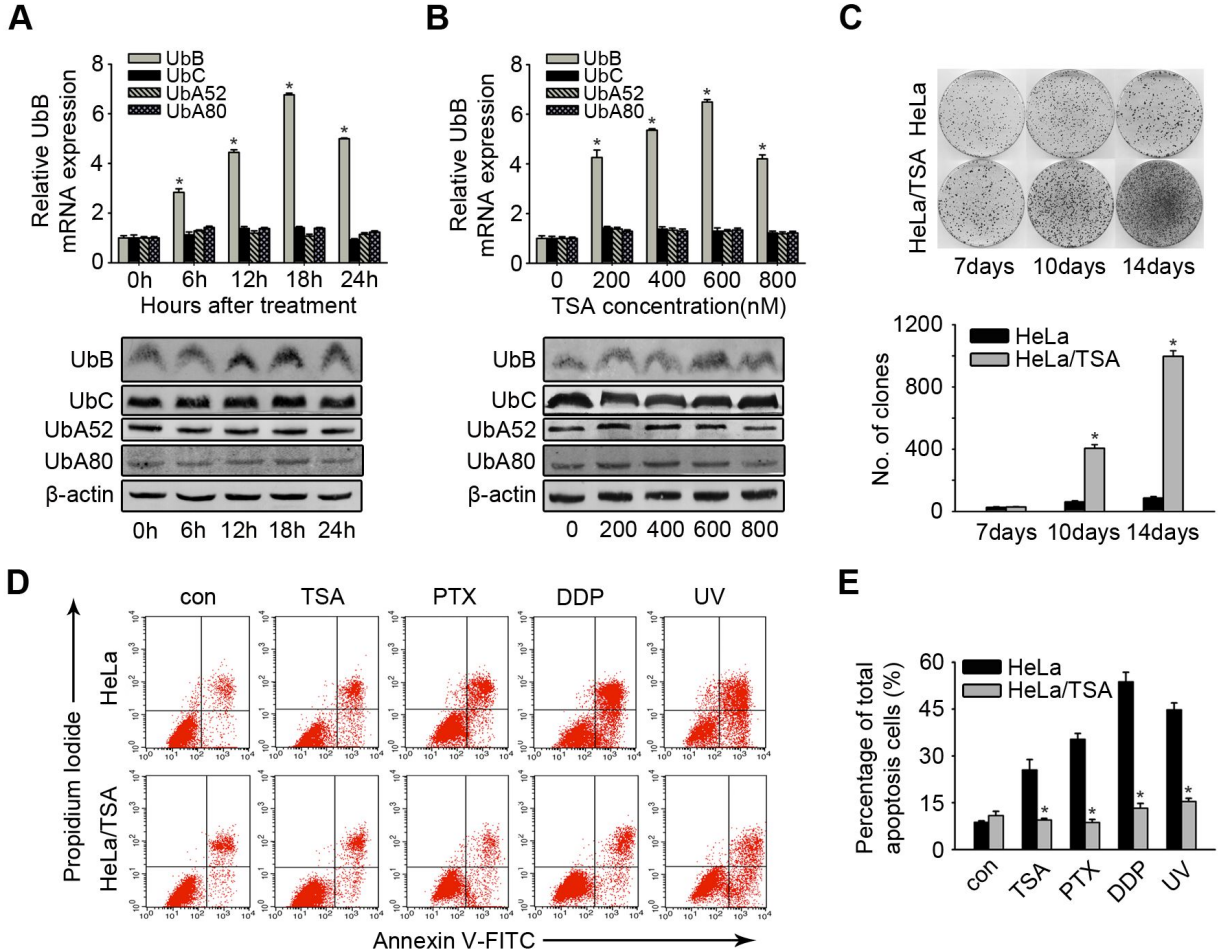
The increasing effect of colony formation and drug-resistance in HeLa/TSA. **A**, HeLa cells were treated with 500 nM TSA for the indicated times and subjected to analysis the mRNA level of UbB, UBC, UbA52 and UbA80 by real-time PCR and the corresponding protein level by western blotting. **B**, HeLa cells were incubated with TSA for 24 hours at a dose ranging from 200 to 800 nM and subjected to analysis the mRNA level of UbB, UbC, UbA52 and UbA80 by real-time PCR and the corresponding protein level by western blotting. **C**, Upper panel: representative dishes of the colony forming assay at day 7, 10 and 14. Lower panel: numbers of colony formed in HeLa and HeLa/TSA at day 7, 10 and 14. The columns represent the average of three separate experiments; error bars, SD; *, *p*<0.05. **D**, HeLa/TSA cells were treated with TSA (1 μM), DDP (30 μM), and PTX (75 nM) for 48 h or UV, the apoptosis cells were quantified by Annexin V/PI staining and the flow cytometry analysis. The representative examples of the flow cytometry results were shown. **E**, Cells were treated as described in **D**, the average percentages of apoptosis cells were reported in the graphs. Values, mean percentages; error bars, SD; *, *p* < 0.05 (n = 3 replications).

The authors confirm that these changes do not alter their findings. The authors have provided the underlying images for all figures in the original article as Supporting Information.

## Supporting Information

S1 File Underlying images for all figures. (ZIP)
