# Supplementary material for: Correction: Ubiquitin B in Cervical Cancer: Critical for the Maintenance of Cancer Stem-Like Cell Characters
Source: PLoS One. 2016 Mar 28;11(3):e0152813. doi: 10.1371/journal.pone.0152813 (PMC4809553; doi:10.1371/journal.pone.0152813)
Supplement: S1 File — (ZIP) [file pone.0152813.s001.zip › underlying images for plos one/Fig 1/Fig.1D apoptosis HeLa-TSA-front 4 panels.pdf]

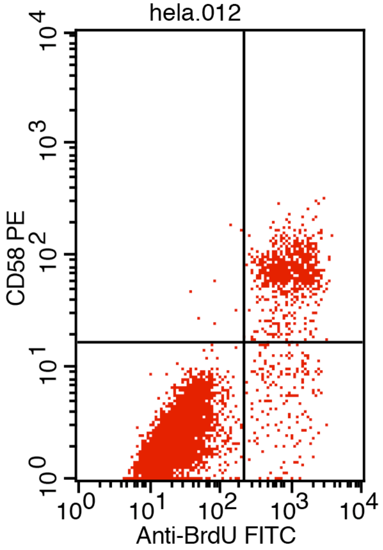

control

File: hela.012

Quad	% Gated
UL	0.09
UR	9.09
LL	89.00
LR	1.82

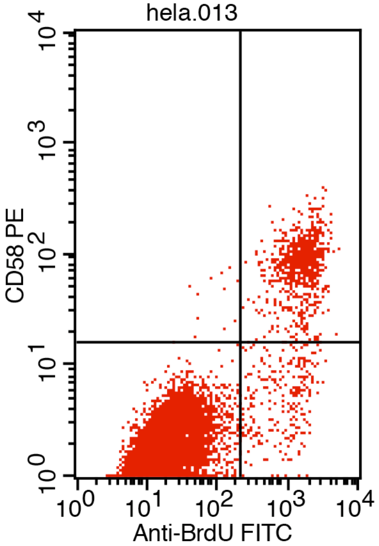

TSA

File: hela.013

Quad	% Gated
UL	0.13
UR	9.52
LL	87.25
LR	3.10

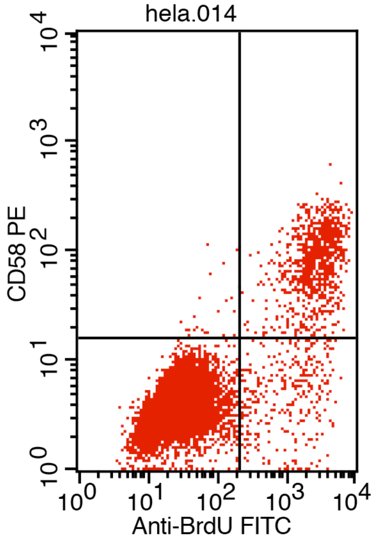

DDP

File: hela.014

Quad	% Gated
UL	0.26
UR	10.08
LL	86.03
LR	3.62

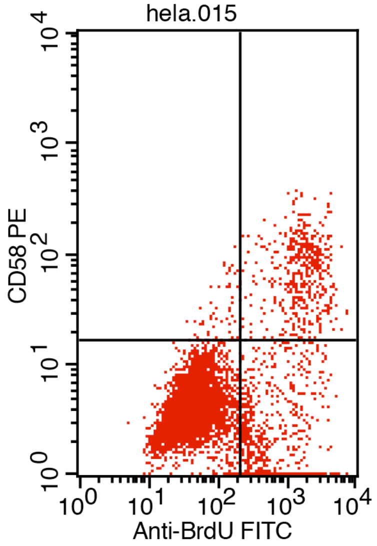

Taxol

File: hela.015

Quad	% Gated
UL	0.62
UR	5.47
LL	83.43
LR	10.49
