# Supplementary material for: Correction: Ubiquitin B in Cervical Cancer: Critical for the Maintenance of Cancer Stem-Like Cell Characters
Source: PLoS One. 2016 Mar 28;11(3):e0152813. doi: 10.1371/journal.pone.0152813 (PMC4809553; doi:10.1371/journal.pone.0152813)
Supplement: S1 File — (ZIP) [file pone.0152813.s001.zip › underlying images for plos one/Fig 1/Fig1.D apoptosis HeLa-front 4 panels.pdf]

HeLa

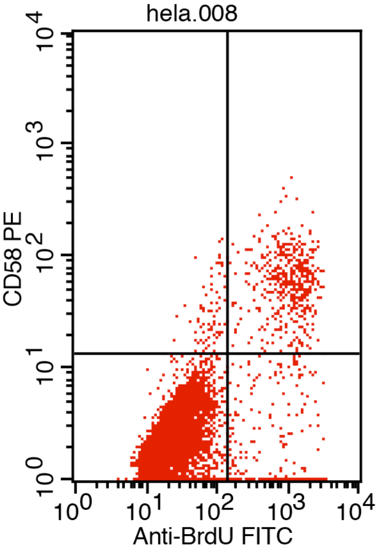

control

File: hela.008

Quad	% Gated
UL	0.86
UR	5.28
LL	90.29
LR	3.57

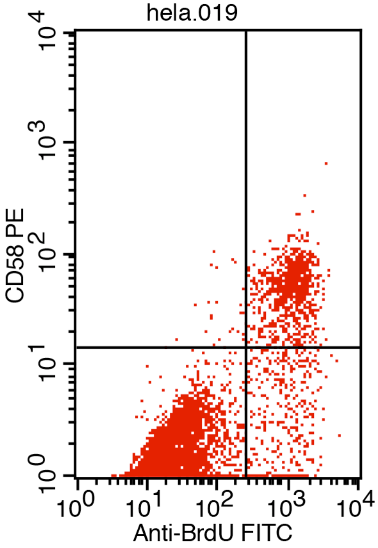

TSA

File: hela.019

Quad	% Gated
UL	0.54
UR	17.69
LL	74.01
LR	7.76

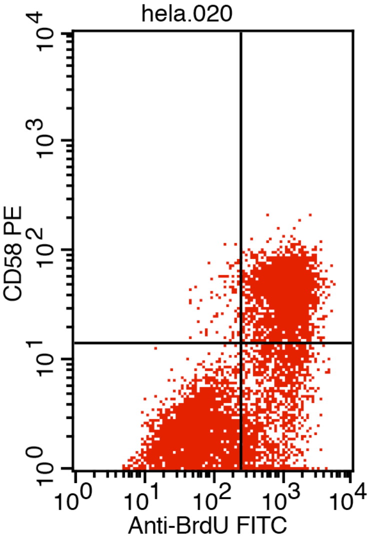

DDP

File: hela.020

Quad	% Gated
UL	1.06
UR	36.32
LL	45.22
LR	17.40

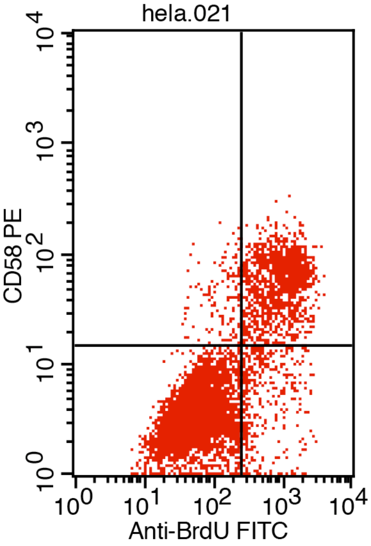

Taxol

File: hela.021

Quad	% Gated
UL	1.96
UR	18.57
LL	73.40
LR	6.08
