# Supplementary figures and images for: Correction: Ubiquitin B in Cervical Cancer: Critical for the Maintenance of Cancer Stem-Like Cell Characters
Source: PLoS One. 2016 Mar 28;11(3):e0152813. doi: 10.1371/journal.pone.0152813 (PMC4809553; doi:10.1371/journal.pone.0152813)

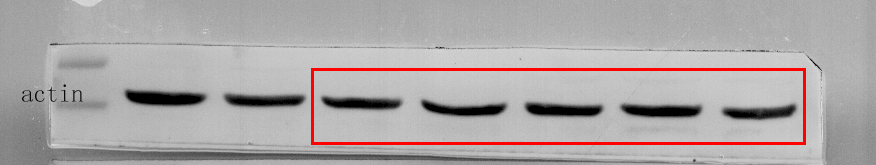

Supplement: S1 File — (ZIP) [file pone.0152813.s001.zip › underlying images for plos one/Fig 1/Fig.1A-actin.jpg]

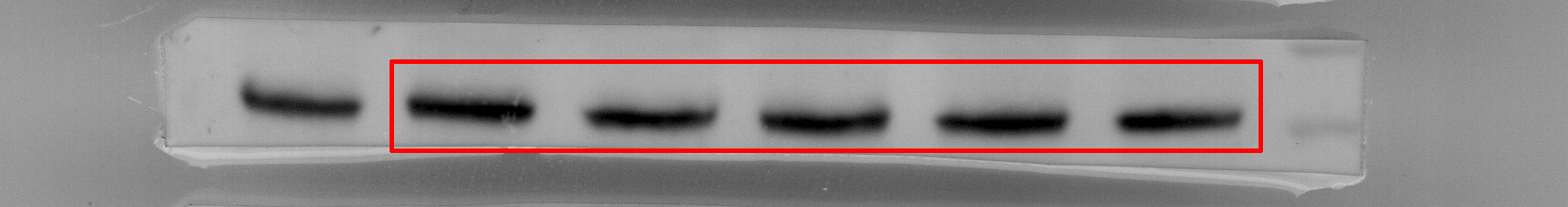

Supplement: S1 File — (ZIP) [file pone.0152813.s001.zip › underlying images for plos one/Fig 1/Fig.1A-UbA52.jpg]

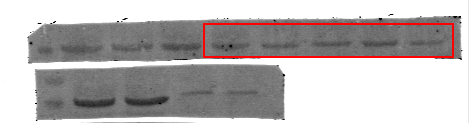

Supplement: S1 File — (ZIP) [file pone.0152813.s001.zip › underlying images for plos one/Fig 1/Fig.1A-UbA80.jpg]

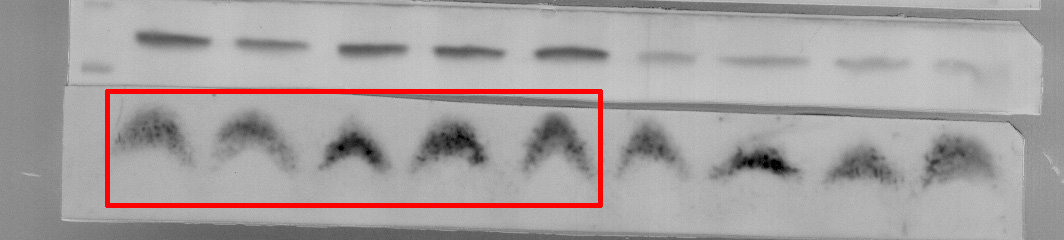

Supplement: S1 File — (ZIP) [file pone.0152813.s001.zip › underlying images for plos one/Fig 1/Fig.1A-UbB.jpg]

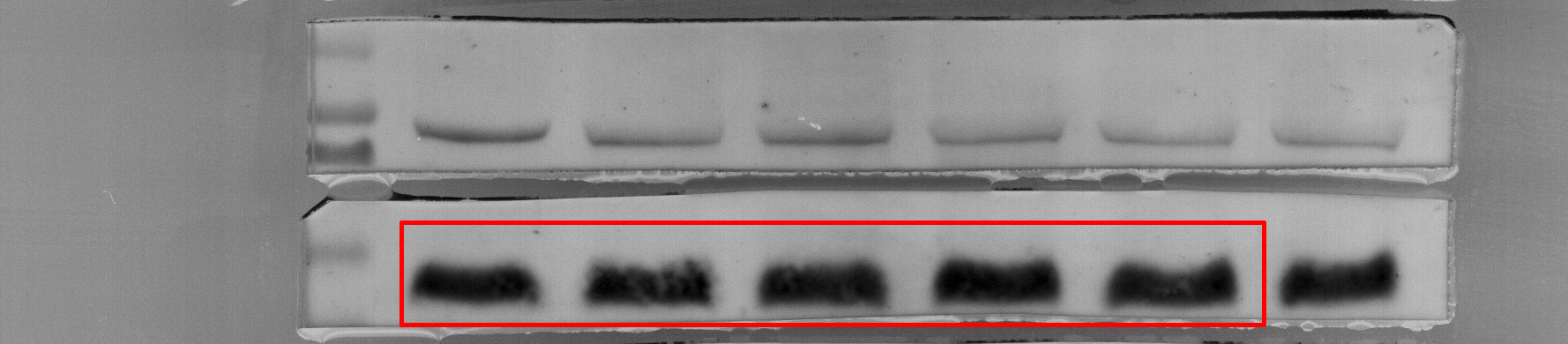

Supplement: S1 File — (ZIP) [file pone.0152813.s001.zip › underlying images for plos one/Fig 1/Fig.1A-UbC.jpg]

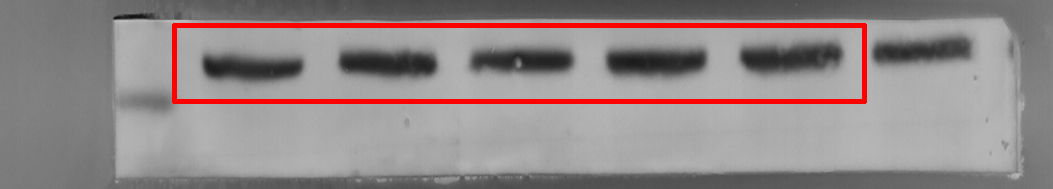

Supplement: S1 File — (ZIP) [file pone.0152813.s001.zip › underlying images for plos one/Fig 1/Fig.1B-actin.jpg]

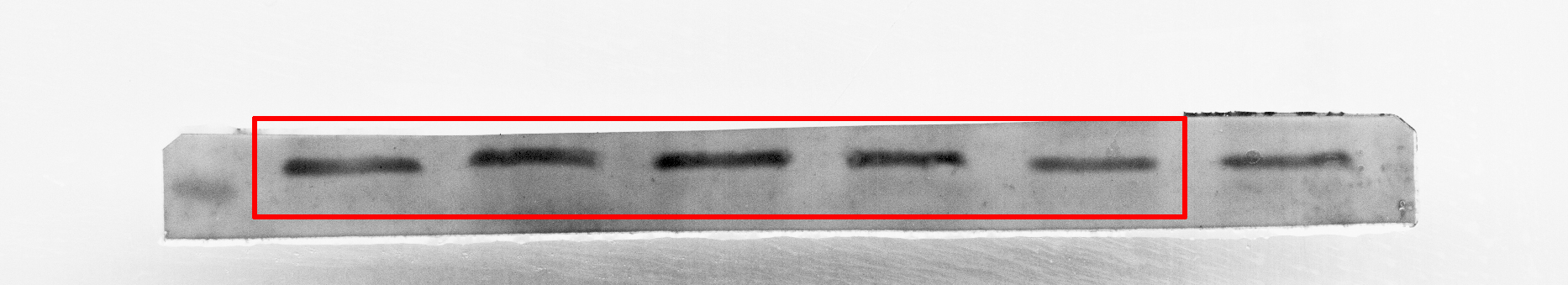

Supplement: S1 File — (ZIP) [file pone.0152813.s001.zip › underlying images for plos one/Fig 1/Fig.1B-UbA52.jpg]

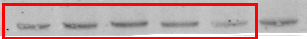

Supplement: S1 File — (ZIP) [file pone.0152813.s001.zip › underlying images for plos one/Fig 1/Fig.1B-UbA80.jpg]

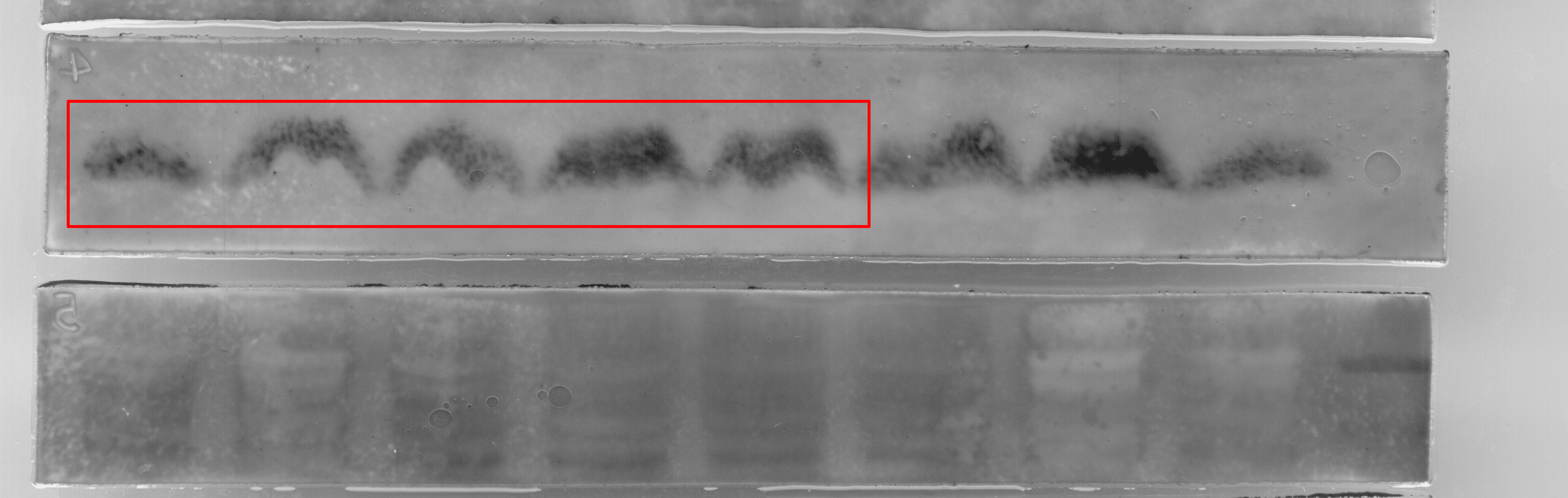

Supplement: S1 File — (ZIP) [file pone.0152813.s001.zip › underlying images for plos one/Fig 1/Fig.1B-UbB.jpg]

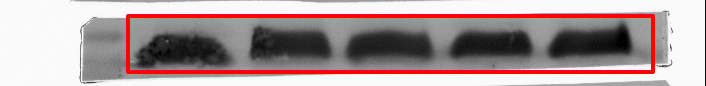

Supplement: S1 File — (ZIP) [file pone.0152813.s001.zip › underlying images for plos one/Fig 1/Fig.1B-UbC.jpg]

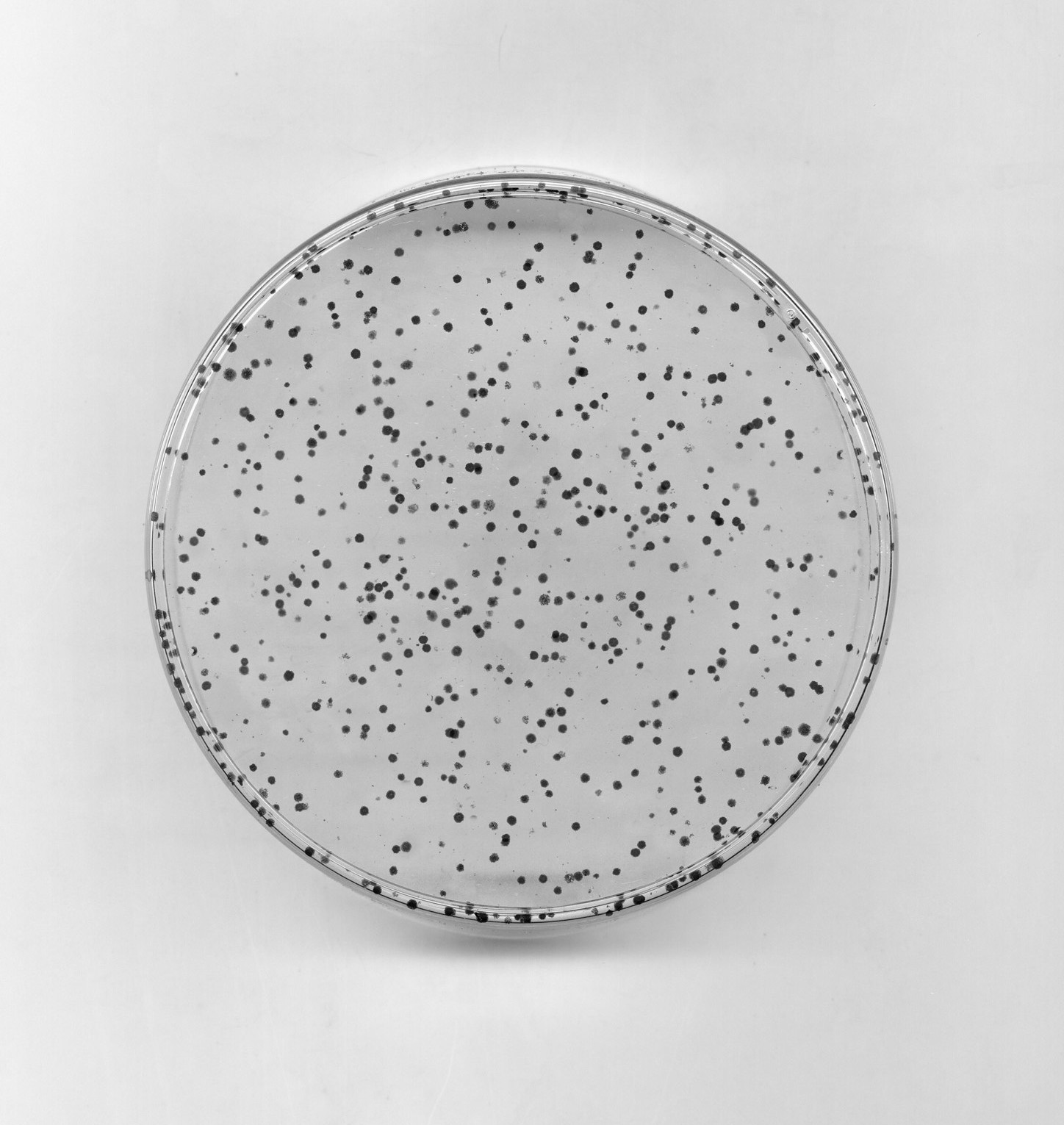

Supplement: S1 File — (ZIP) [file pone.0152813.s001.zip › underlying images for plos one/Fig 1/Fig.1C-HeLa-10days.jpg]

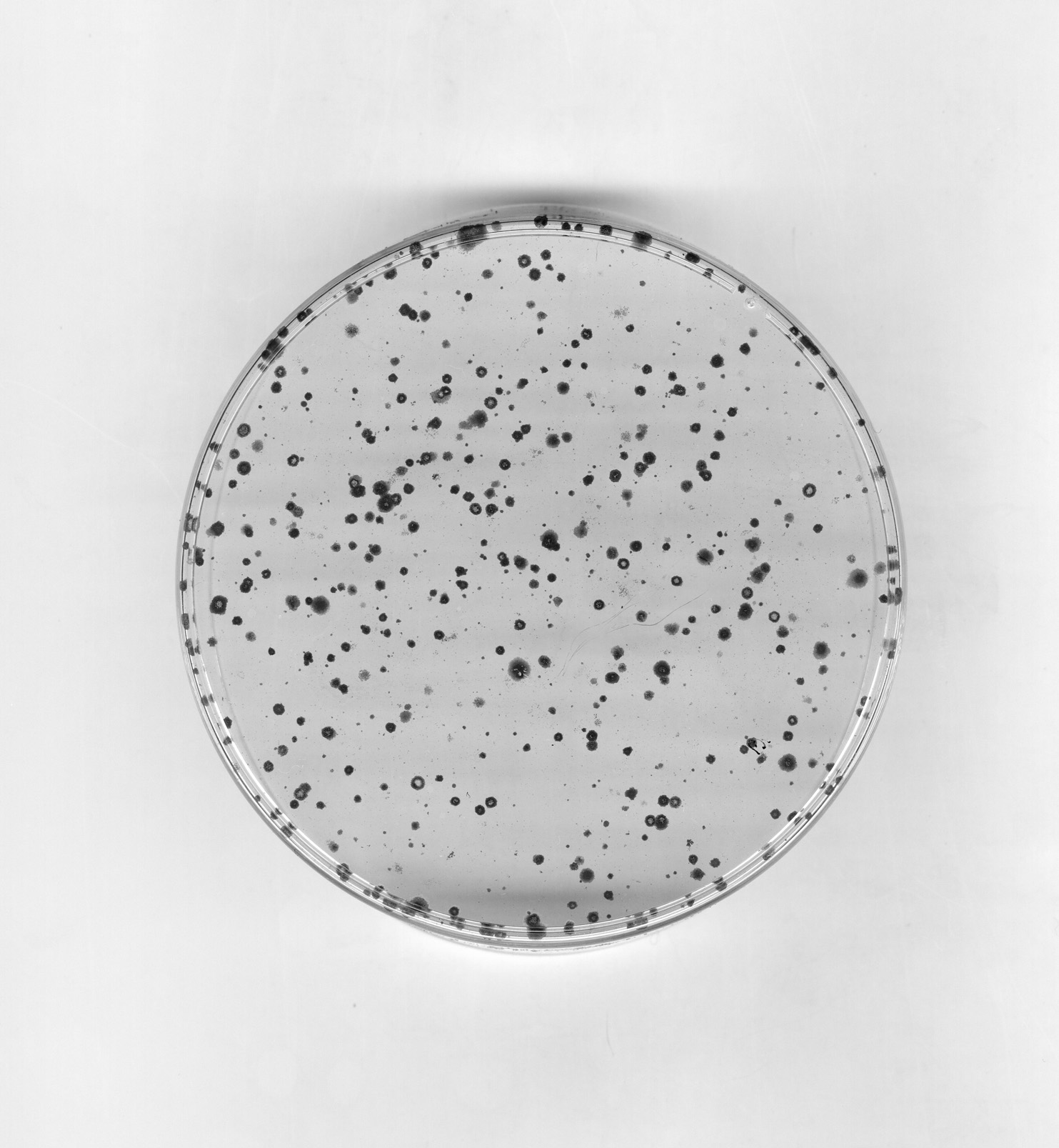

Supplement: S1 File — (ZIP) [file pone.0152813.s001.zip › underlying images for plos one/Fig 1/Fig.1C-HeLa-14days.jpg]

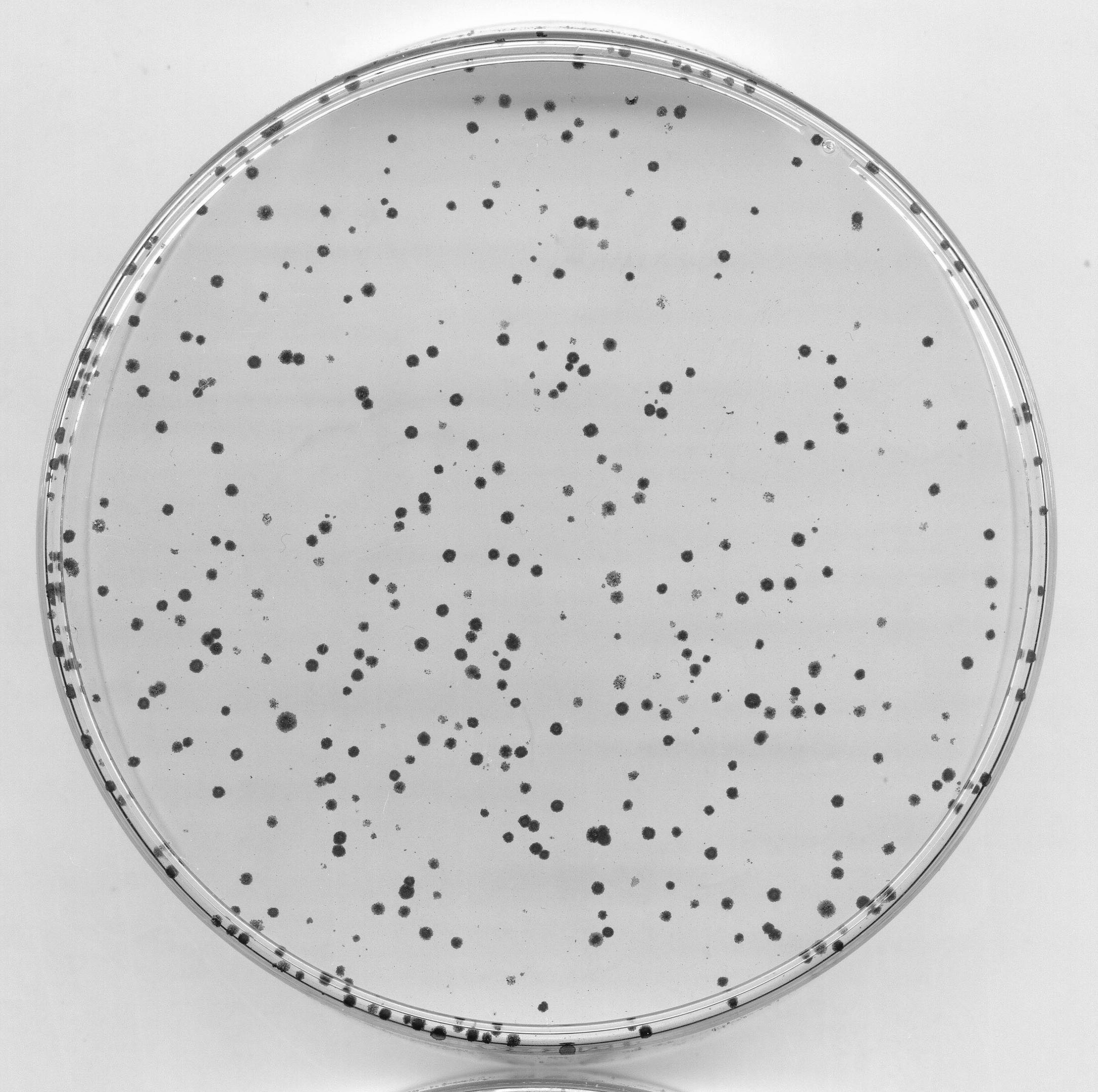

Supplement: S1 File — (ZIP) [file pone.0152813.s001.zip › underlying images for plos one/Fig 1/Fig.1C-HeLa-7days.jpg]

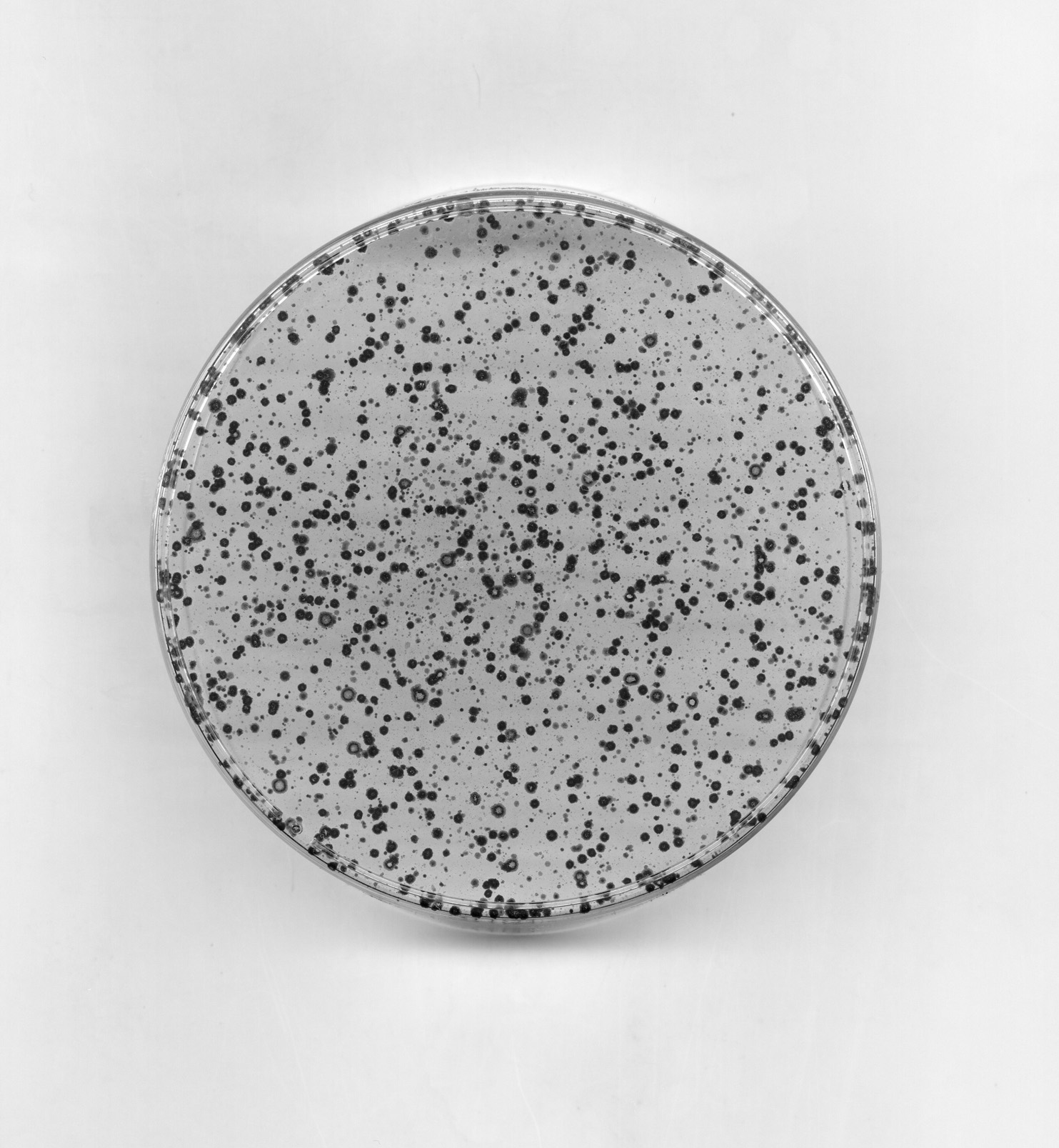

Supplement: S1 File — (ZIP) [file pone.0152813.s001.zip › underlying images for plos one/Fig 1/Fig.1C-HeLa-TSA-10days.jpg]

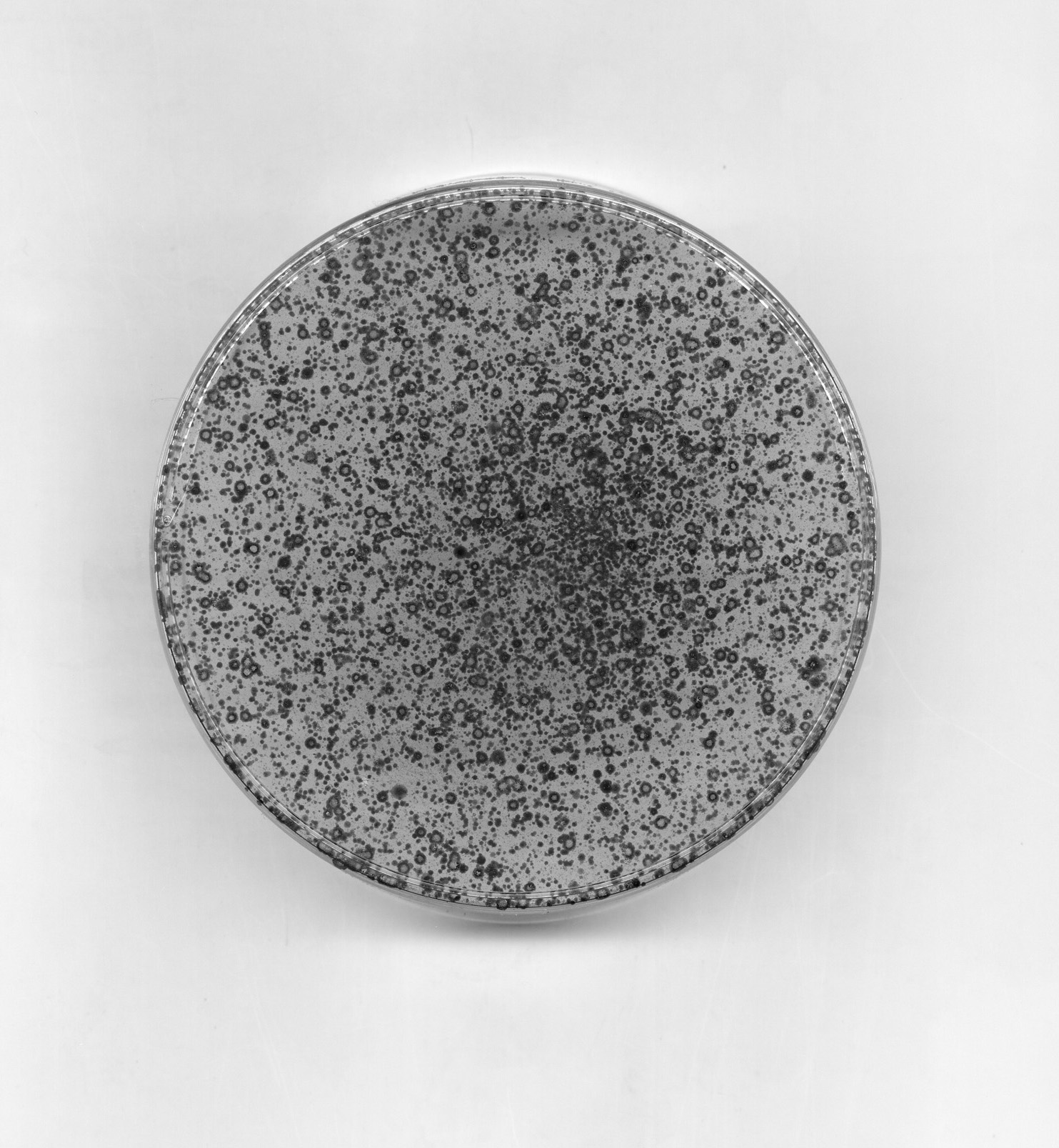

Supplement: S1 File — (ZIP) [file pone.0152813.s001.zip › underlying images for plos one/Fig 1/Fig.1C-Hela-TSA-14days.jpg]

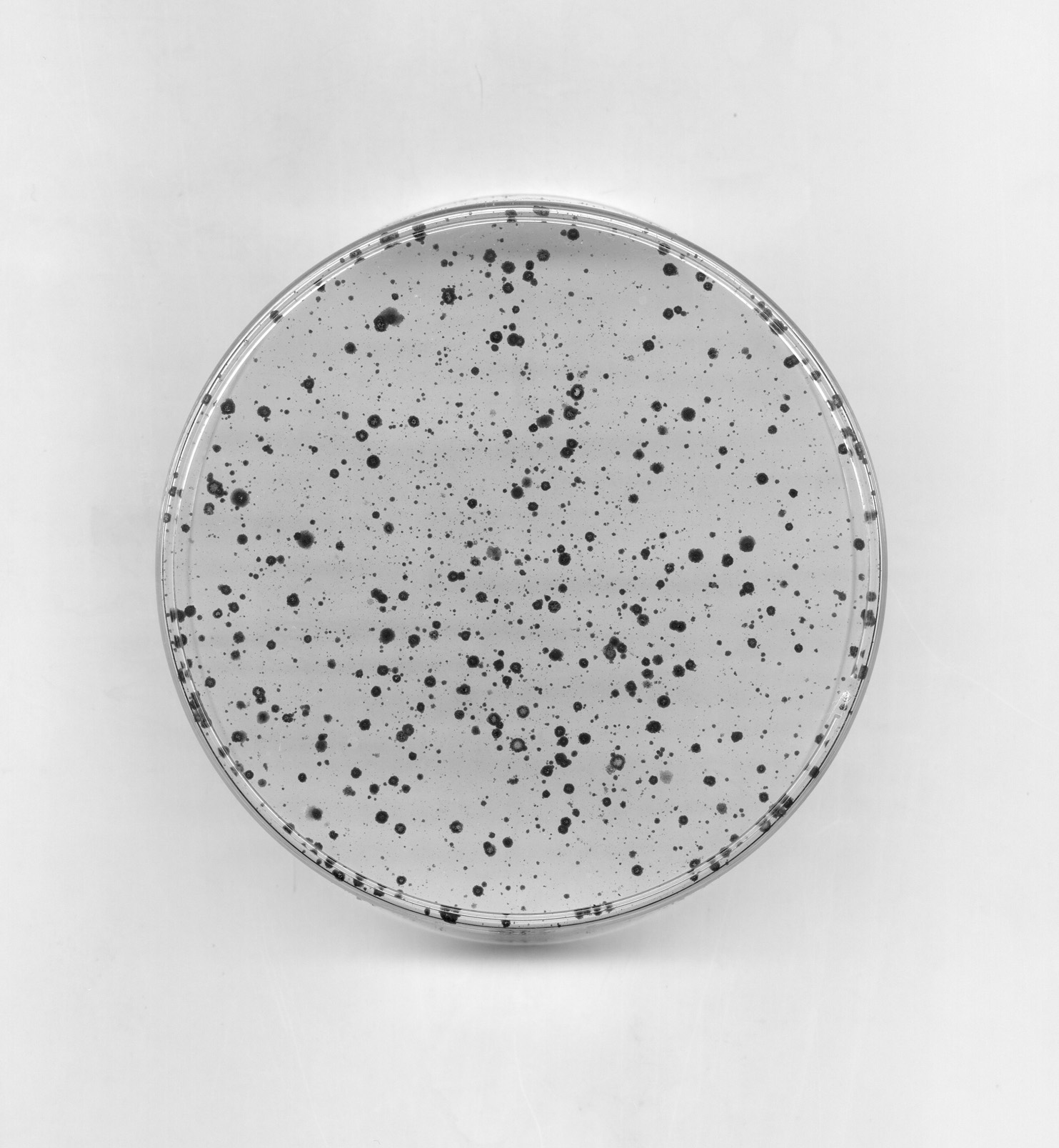

Supplement: S1 File — (ZIP) [file pone.0152813.s001.zip › underlying images for plos one/Fig 1/Fig.1C-HeLa-TSA-7days.jpg]

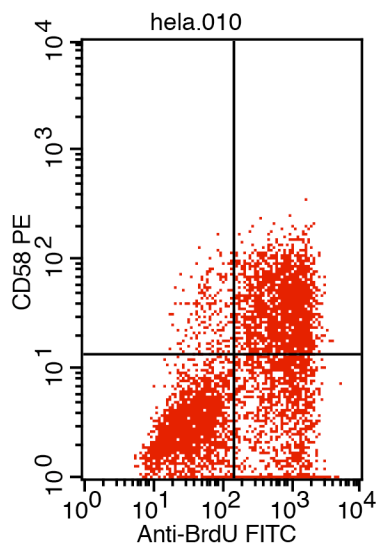

File: hela.010

HeLa  
+UV

| Quad | % Gated |
|------|---------|
| UL   | 2.69    |
| UR   | 28.53   |
| LL   | 33.84   |
| LR   | 34.95   |

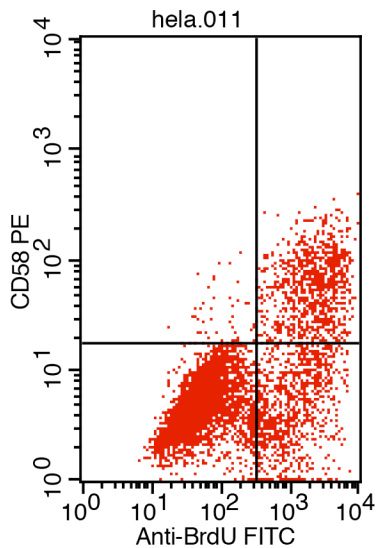

File: hela.011

HeLa/TSA  
+UV

| Quad | % Gated |
|------|---------|
| UL   | 0.72    |
| UR   | 12.09   |
| LL   | 75.24   |
| LR   | 11.96   |

Supplement: S1 File — (ZIP) [file pone.0152813.s001.zip › underlying images for plos one/Fig 1/Fig.1D apoptosis-UV-HeLa & HeLa-TSA1.pdf]

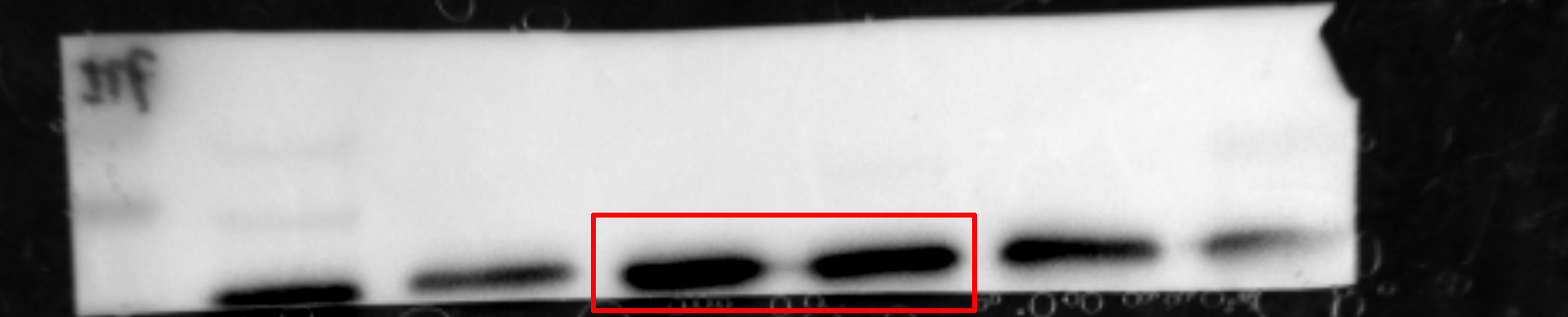

Supplement: S1 File — (ZIP) [file pone.0152813.s001.zip › underlying images for plos one/Fig 2/Fig.2b-actin.jpg]

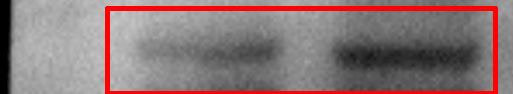

Supplement: S1 File — (ZIP) [file pone.0152813.s001.zip › underlying images for plos one/Fig 2/Fig.2b-Mdr-1.jpg]

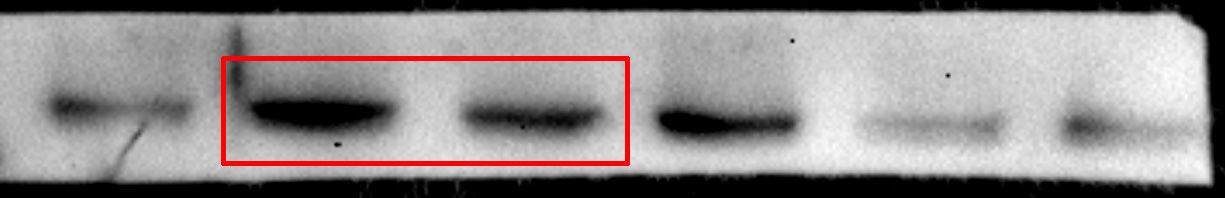

Supplement: S1 File — (ZIP) [file pone.0152813.s001.zip › underlying images for plos one/Fig 2/Fig.2b-pAKT.jpg]

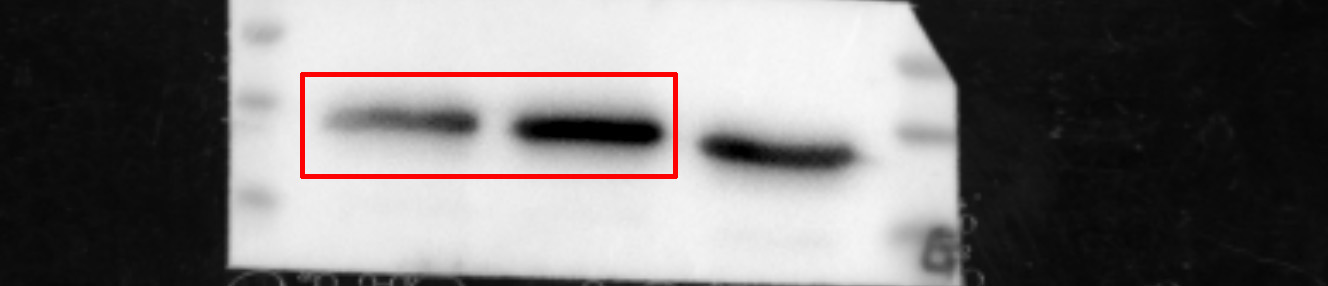

Supplement: S1 File — (ZIP) [file pone.0152813.s001.zip › underlying images for plos one/Fig 2/Fig.2B-pSTAT3.jpg]

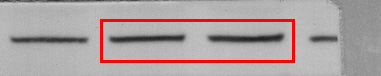

Supplement: S1 File — (ZIP) [file pone.0152813.s001.zip › underlying images for plos one/Fig 2/Fig.2b-UbA52.jpg]

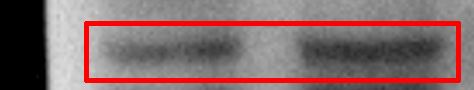

Supplement: S1 File — (ZIP) [file pone.0152813.s001.zip › underlying images for plos one/Fig 2/Fig.2b-UbA80.jpg]

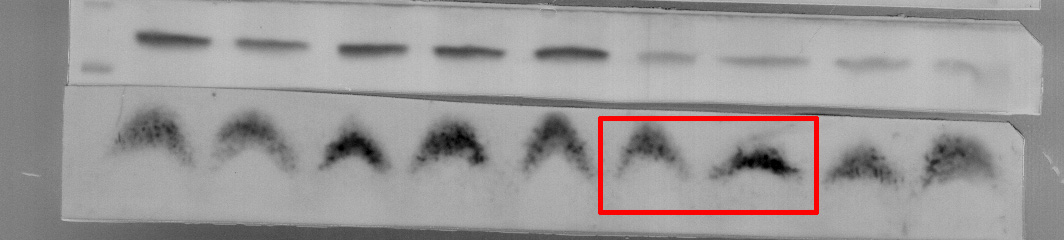

Supplement: S1 File — (ZIP) [file pone.0152813.s001.zip › underlying images for plos one/Fig 2/Fig.2B-UbB.jpg]

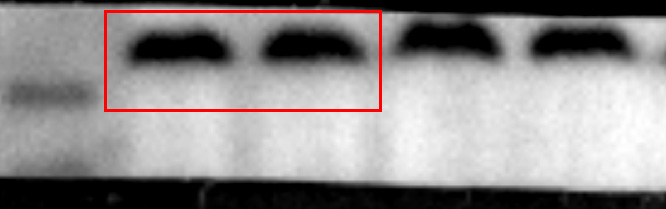

Supplement: S1 File — (ZIP) [file pone.0152813.s001.zip › underlying images for plos one/Fig 2/Fig.2b-UbC.jpg]

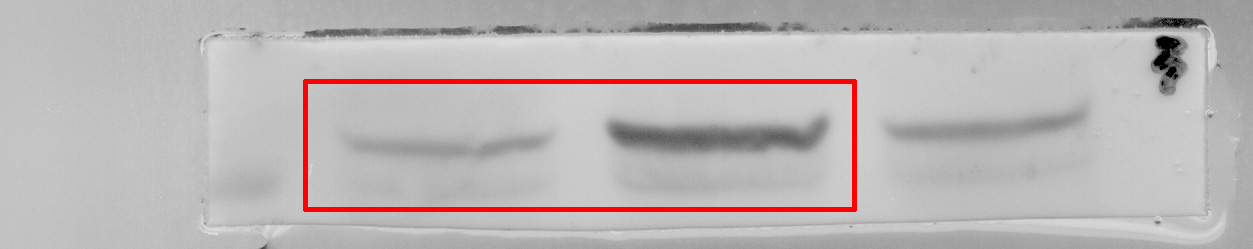

Supplement: S1 File — (ZIP) [file pone.0152813.s001.zip › underlying images for plos one/Fig 2/Fig.2b-vimentin.jpg]

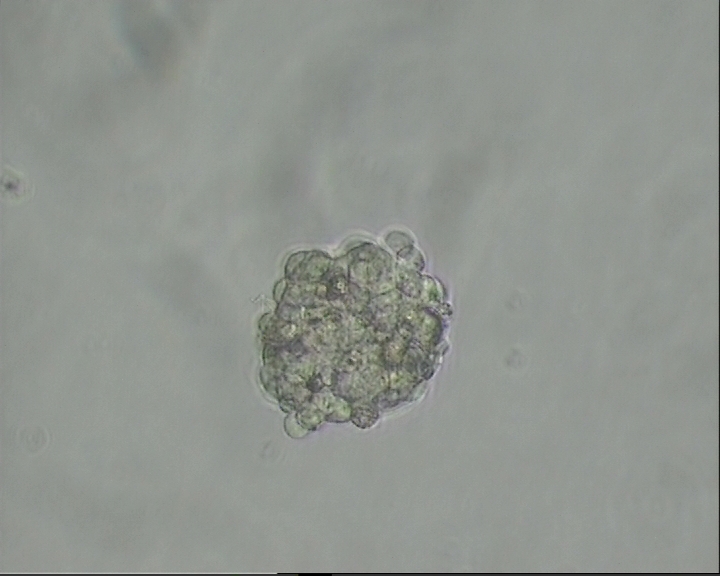

Supplement: S1 File — (ZIP) [file pone.0152813.s001.zip › underlying images for plos one/Fig 2/Fig.2E-HeLa-16days.jpg]

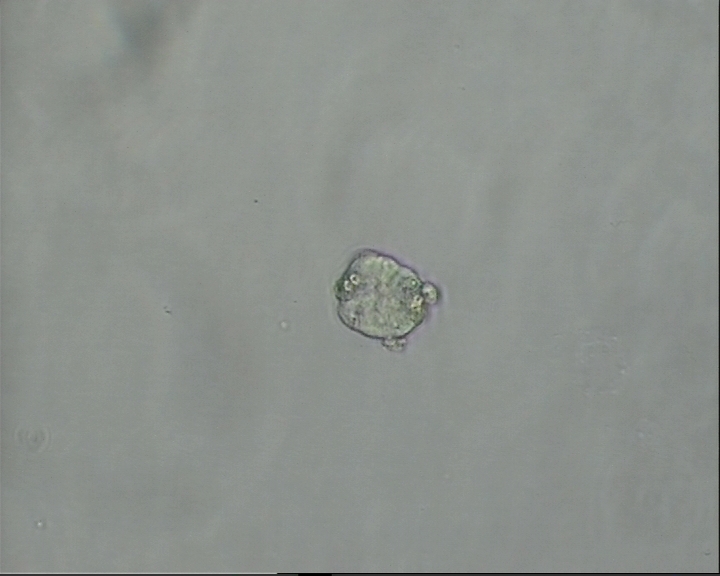

Supplement: S1 File — (ZIP) [file pone.0152813.s001.zip › underlying images for plos one/Fig 2/Fig.2E-HeLa-8days.jpg]

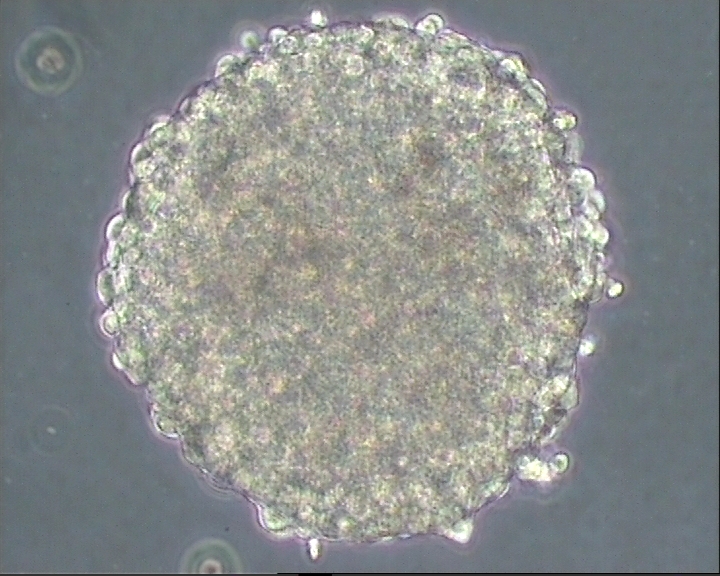

Supplement: S1 File — (ZIP) [file pone.0152813.s001.zip › underlying images for plos one/Fig 2/Fig.2E-HeLa-TSA-16days.jpg]

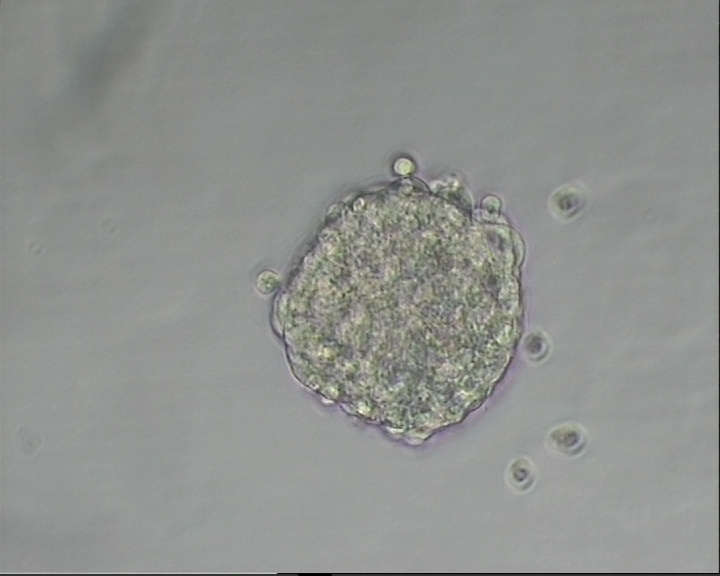

Supplement: S1 File — (ZIP) [file pone.0152813.s001.zip › underlying images for plos one/Fig 2/Fig.2E-HeLa-TSA-8days.jpg]

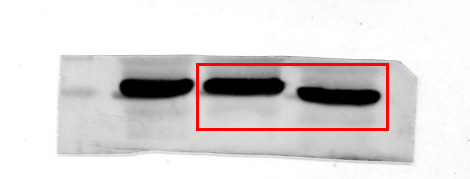

Supplement: S1 File — (ZIP) [file pone.0152813.s001.zip › underlying images for plos one/Fig 3/Fig.3B-actin.jpg]

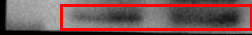

Supplement: S1 File — (ZIP) [file pone.0152813.s001.zip › underlying images for plos one/Fig 3/Fig.3B-Mdr-1.jpg]

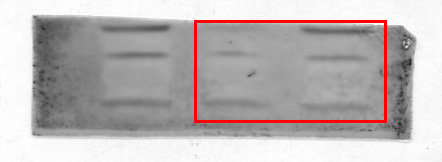

Supplement: S1 File — (ZIP) [file pone.0152813.s001.zip › underlying images for plos one/Fig 3/Fig.3B-Nanog.jpg]

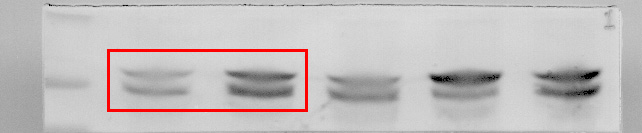

Supplement: S1 File — (ZIP) [file pone.0152813.s001.zip › underlying images for plos one/Fig 3/Fig.3B-Oct4.jpg]

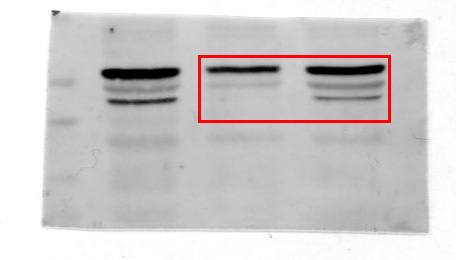

Supplement: S1 File — (ZIP) [file pone.0152813.s001.zip › underlying images for plos one/Fig 3/Fig.3B-Sox2.jpg]

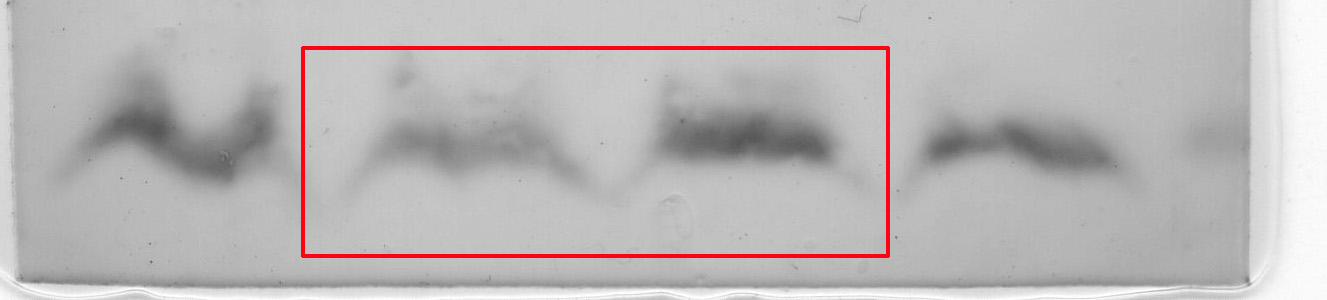

Supplement: S1 File — (ZIP) [file pone.0152813.s001.zip › underlying images for plos one/Fig 3/Fig.3B-UbB.jpg]

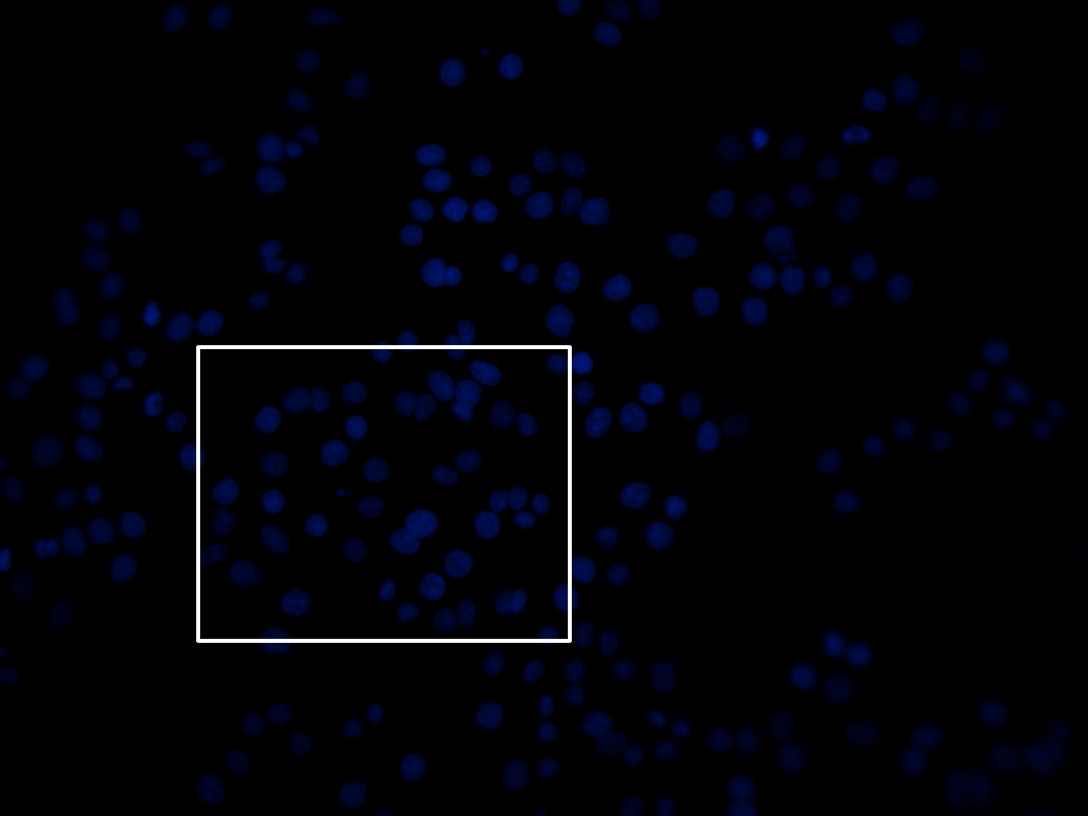

Supplement: S1 File — (ZIP) [file pone.0152813.s001.zip › underlying images for plos one/Fig 3/Fig.3C-HeLa-Nanog-DAPI.jpg]

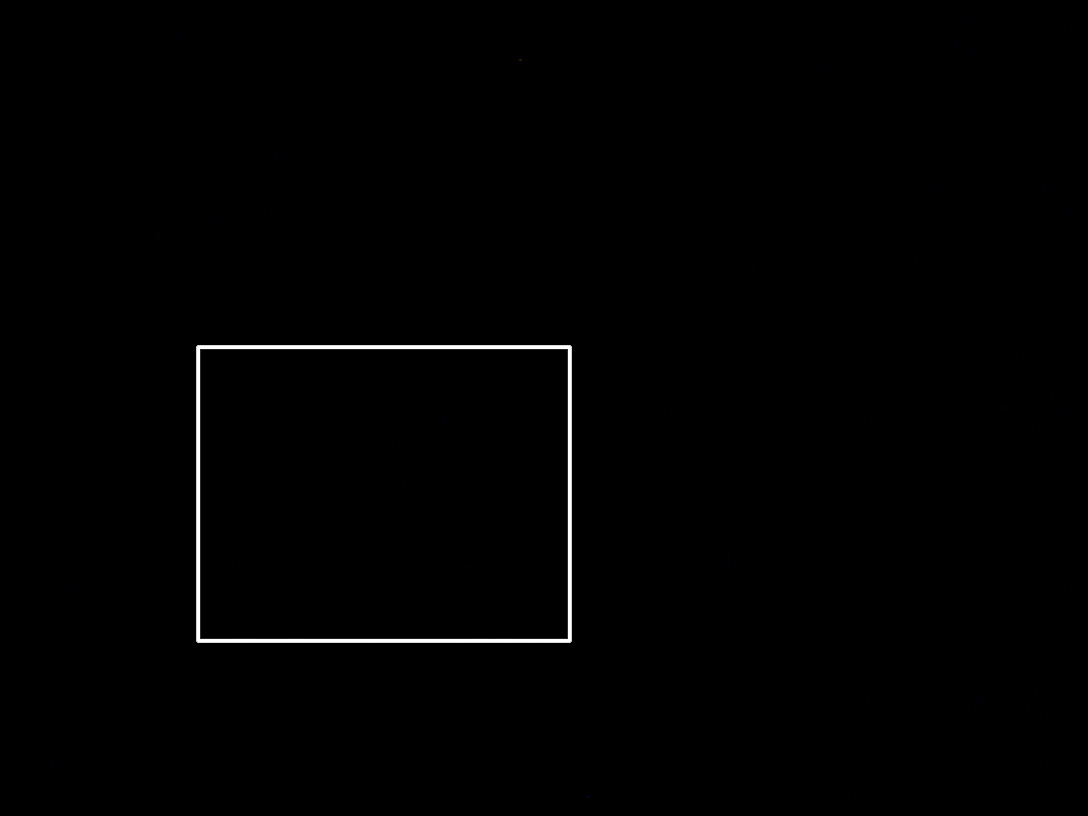

Supplement: S1 File — (ZIP) [file pone.0152813.s001.zip › underlying images for plos one/Fig 3/Fig.3C-HeLa-Nanog.jpg]

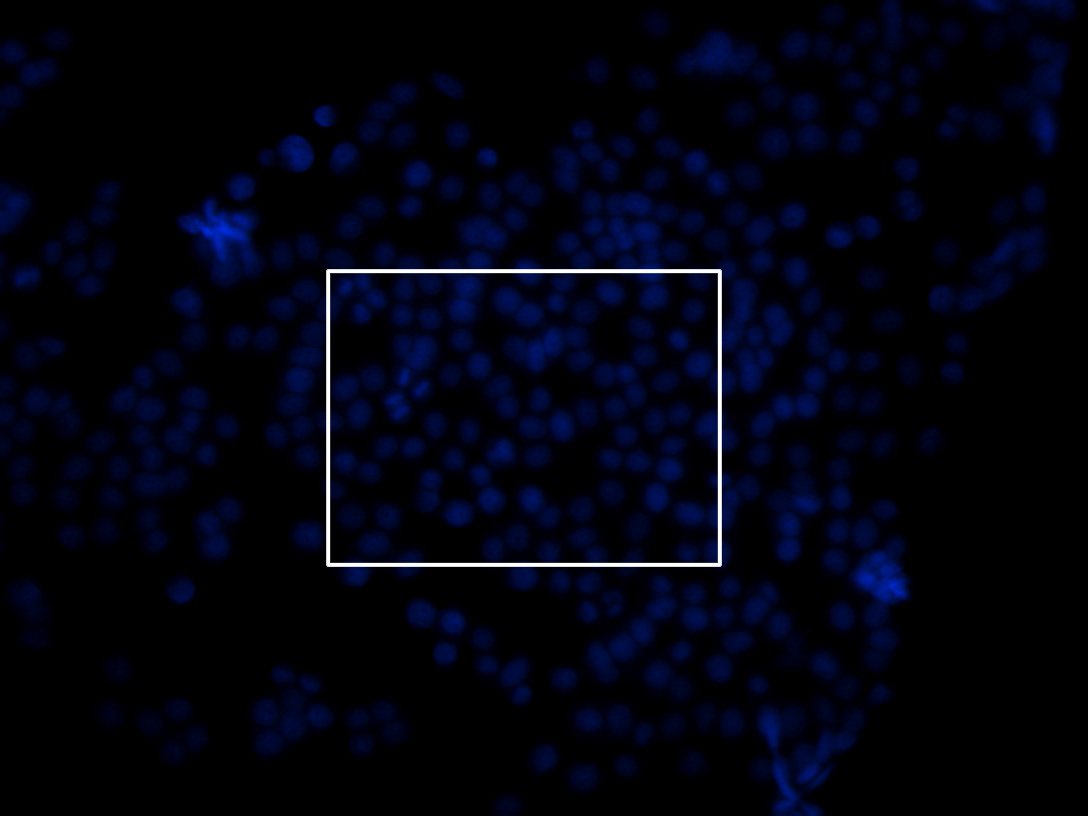

Supplement: S1 File — (ZIP) [file pone.0152813.s001.zip › underlying images for plos one/Fig 3/Fig.3C-HeLa-Oct4-DAPI.jpg]

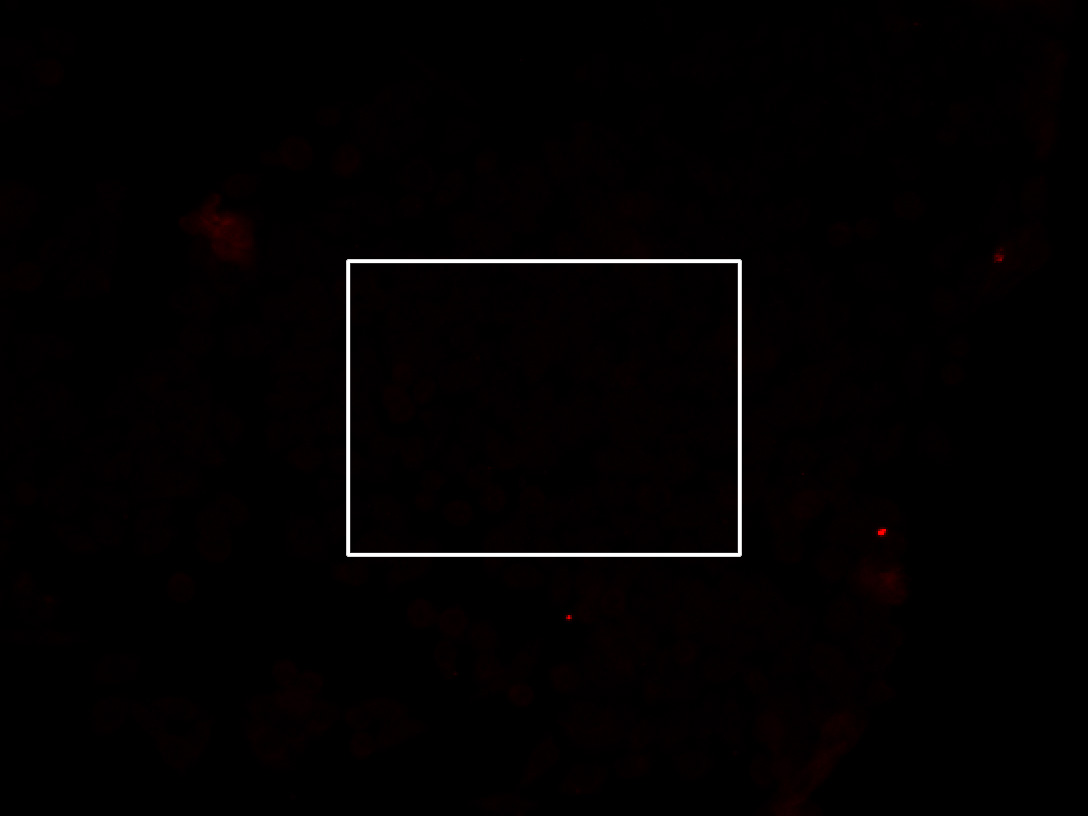

Supplement: S1 File — (ZIP) [file pone.0152813.s001.zip › underlying images for plos one/Fig 3/Fig.3C-HeLa-Oct4.jpg]

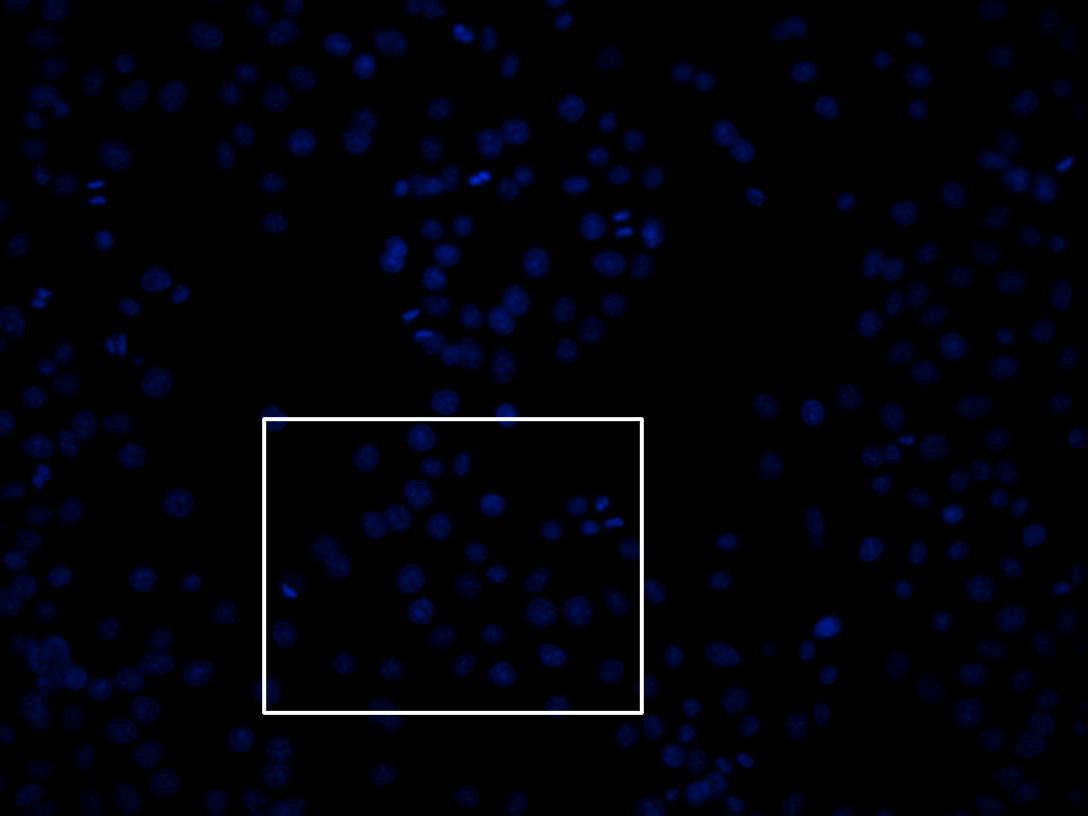

Supplement: S1 File — (ZIP) [file pone.0152813.s001.zip › underlying images for plos one/Fig 3/Fig.3C-HeLa-Sox2-DAPI.jpg]

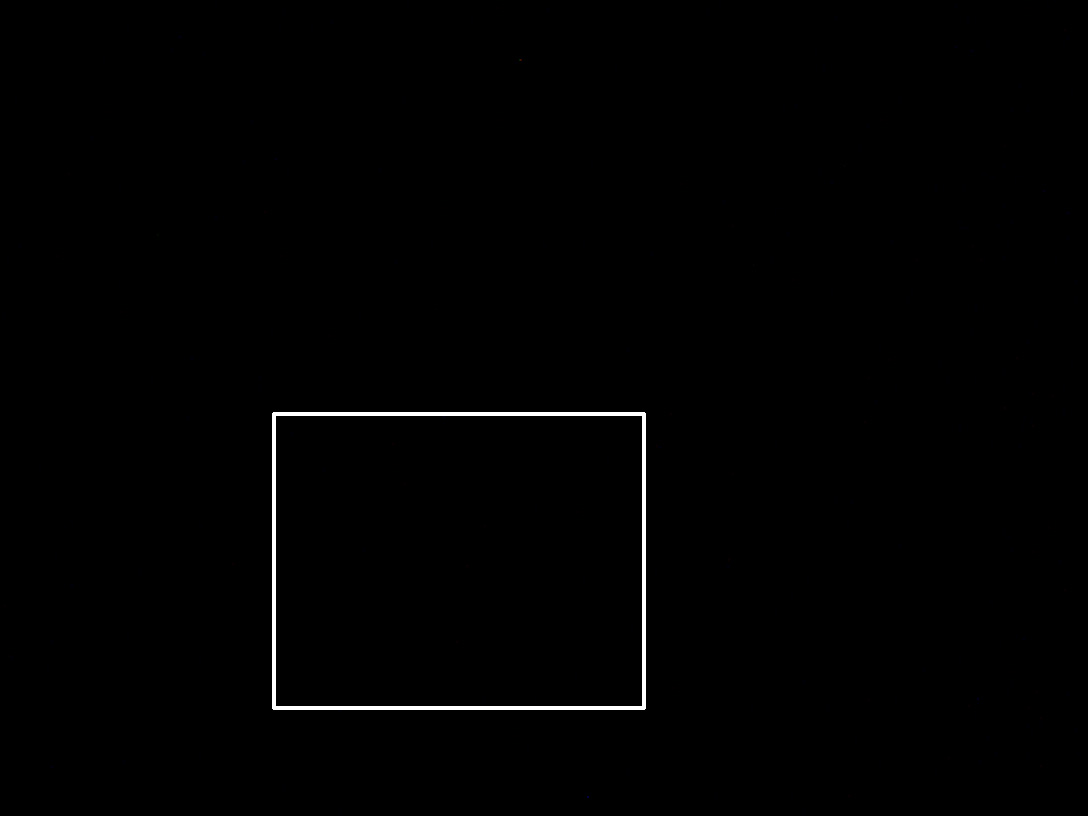

Supplement: S1 File — (ZIP) [file pone.0152813.s001.zip › underlying images for plos one/Fig 3/Fig.3C-HeLa-Sox2.jpg]

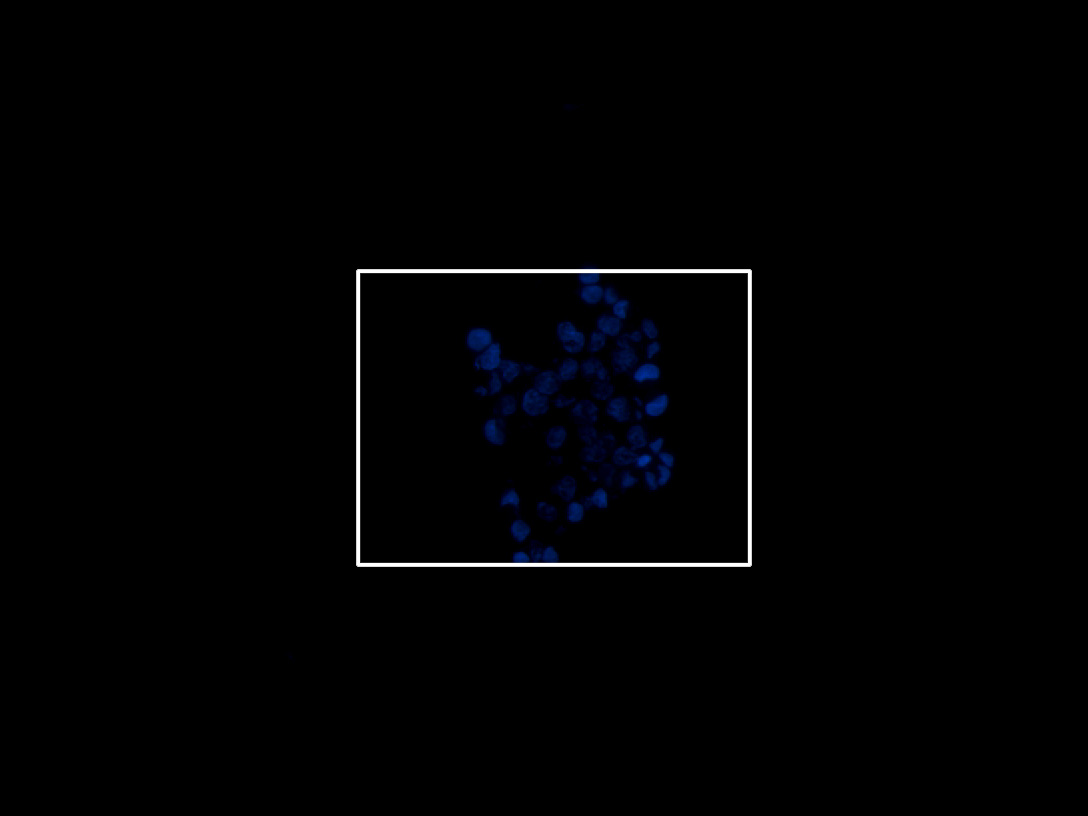

Supplement: S1 File — (ZIP) [file pone.0152813.s001.zip › underlying images for plos one/Fig 3/Fig.3C-HeLa-TSA-Nanog-DAPI.jpg]

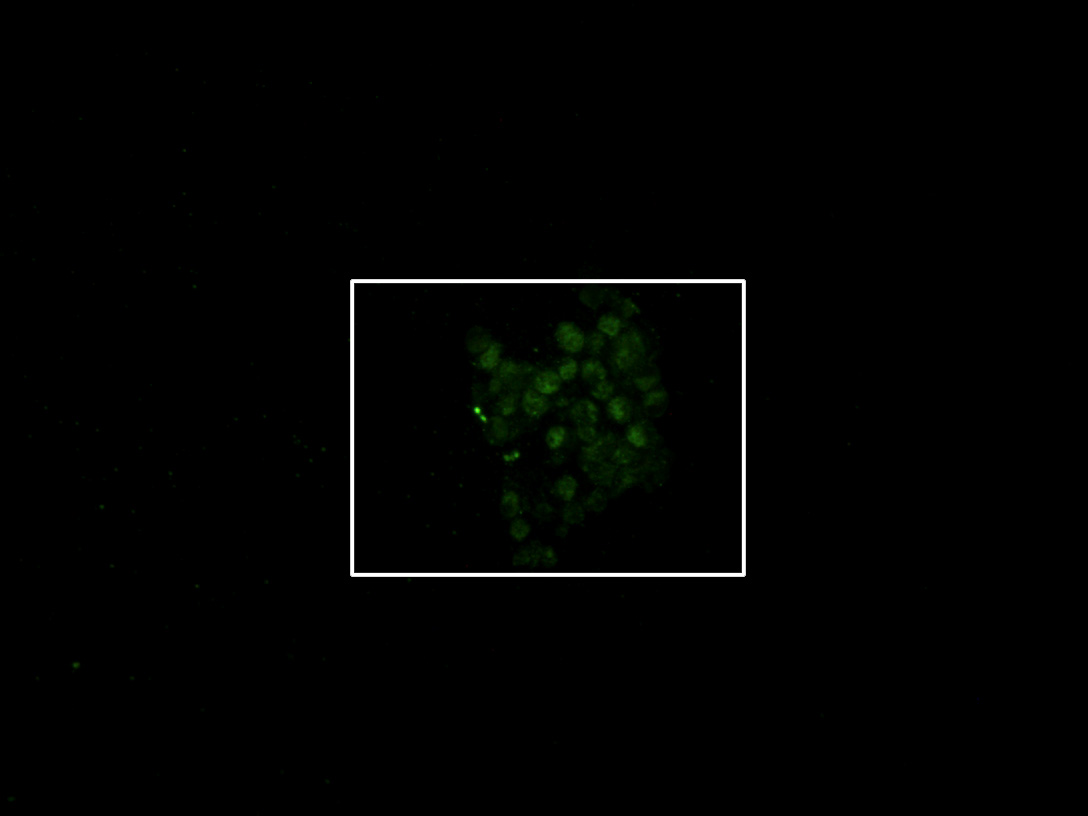

Supplement: S1 File — (ZIP) [file pone.0152813.s001.zip › underlying images for plos one/Fig 3/Fig.3C-HeLa-TSA-Nanog.jpg]

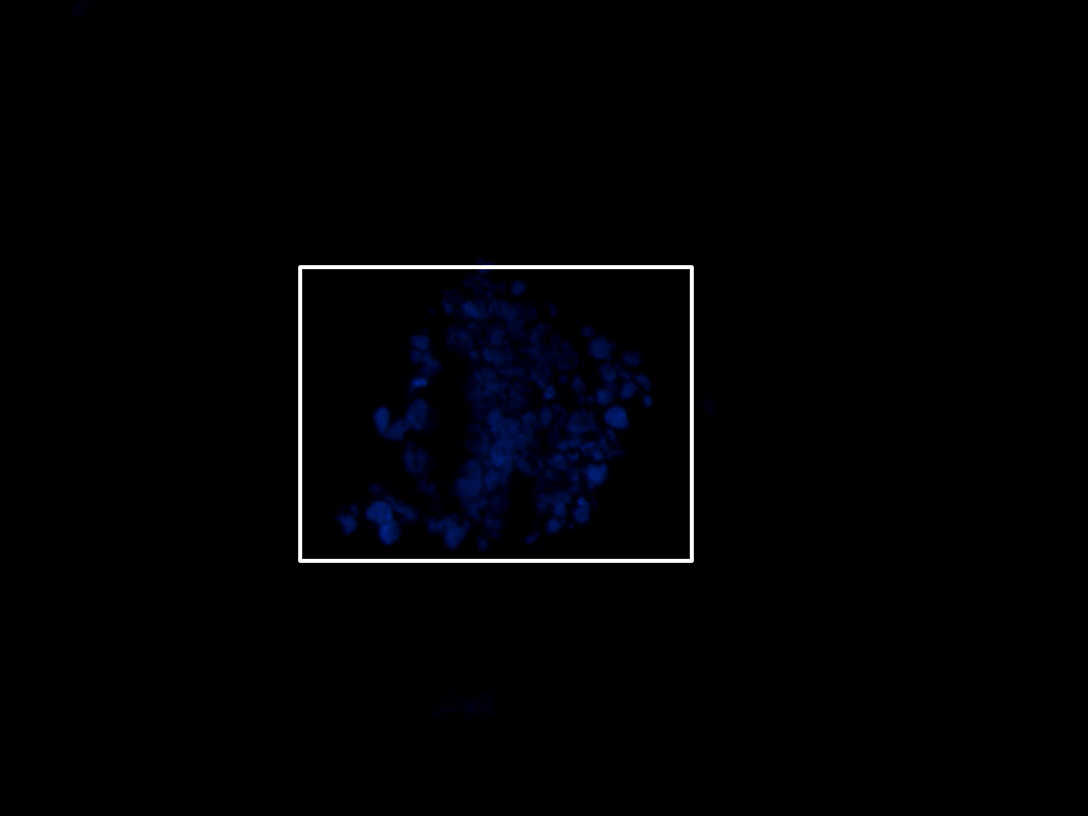

Supplement: S1 File — (ZIP) [file pone.0152813.s001.zip › underlying images for plos one/Fig 3/Fig.3C-HeLa-TSA-Oct4-DAPI.jpg]

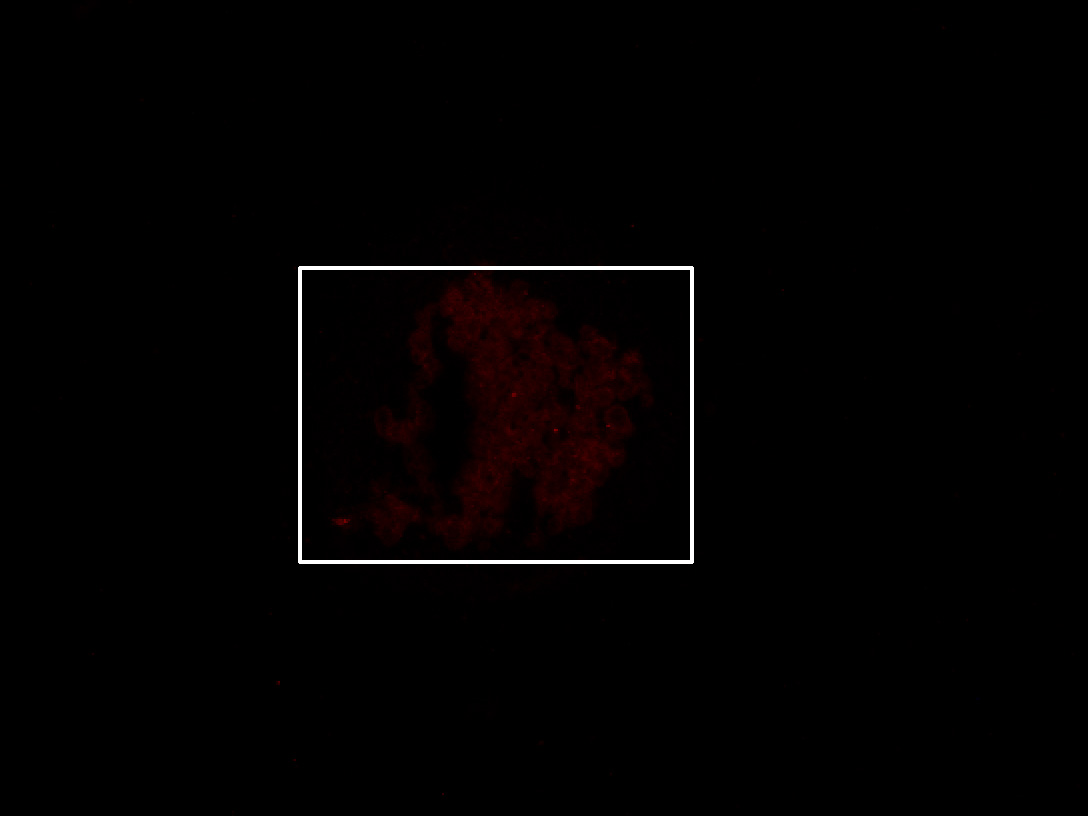

Supplement: S1 File — (ZIP) [file pone.0152813.s001.zip › underlying images for plos one/Fig 3/Fig.3C-HeLa-TSA-Oct4.jpg]

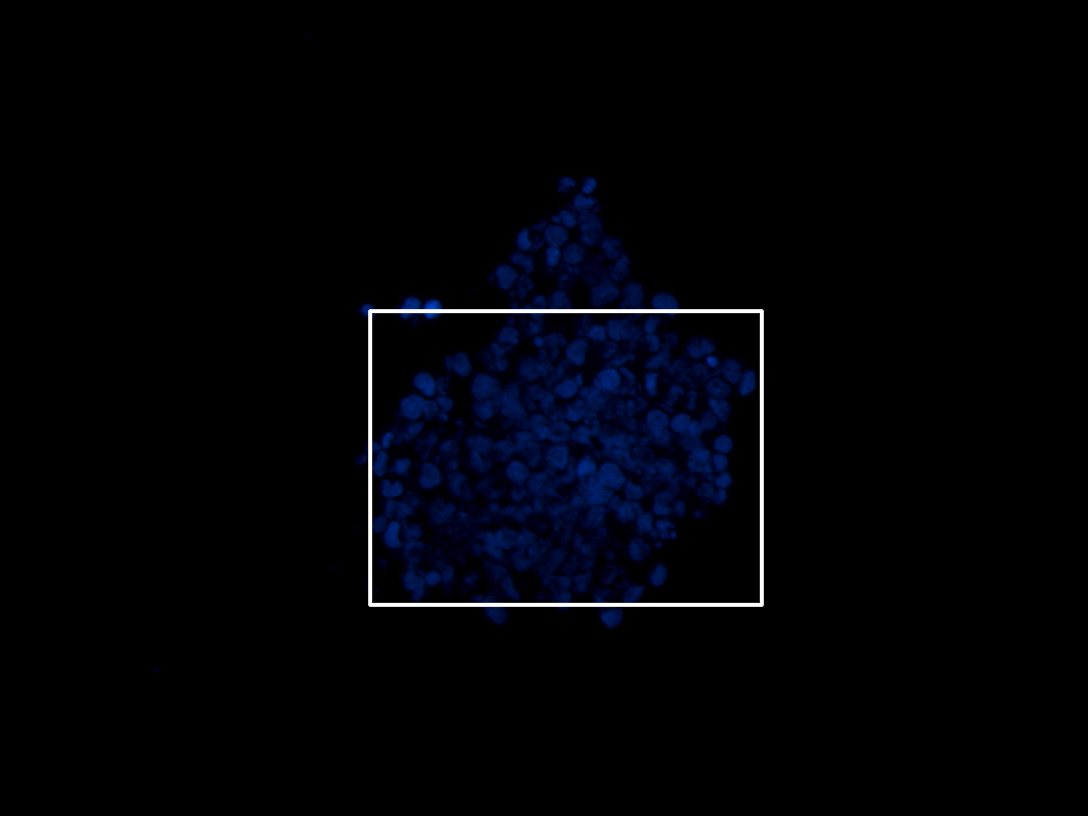

Supplement: S1 File — (ZIP) [file pone.0152813.s001.zip › underlying images for plos one/Fig 3/Fig.3C-HeLa-TSA-Sox2-DAPI.jpg]

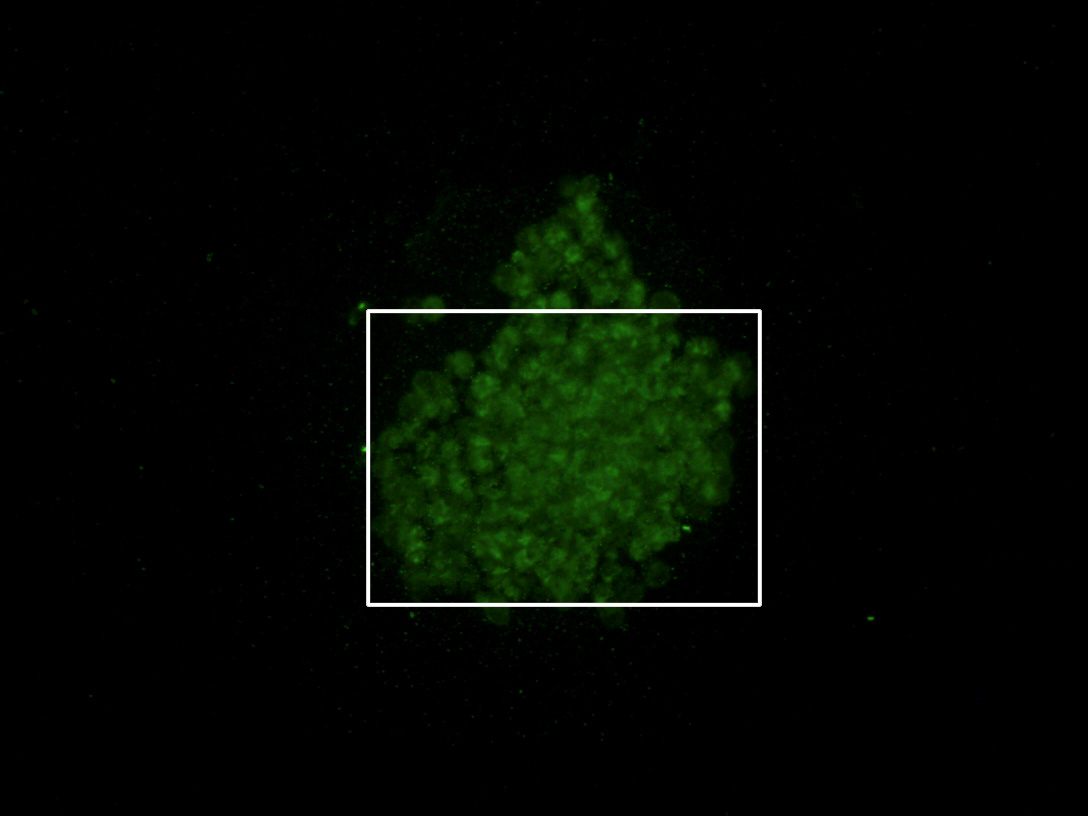

Supplement: S1 File — (ZIP) [file pone.0152813.s001.zip › underlying images for plos one/Fig 3/Fig.3C-HeLa-TSA-Sox2.jpg]

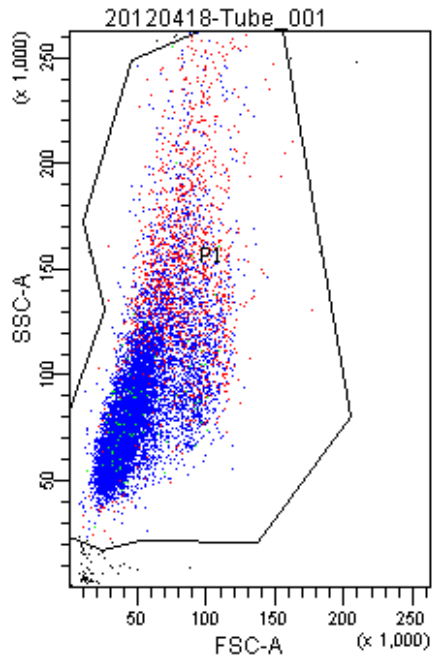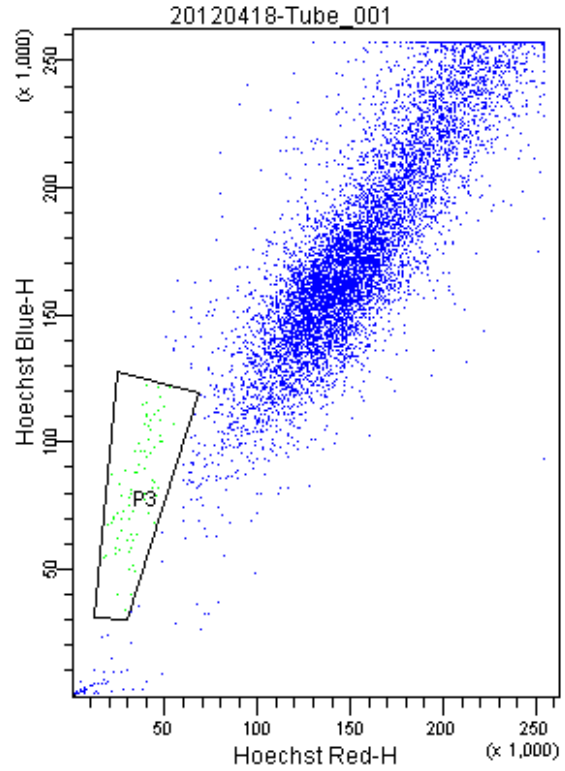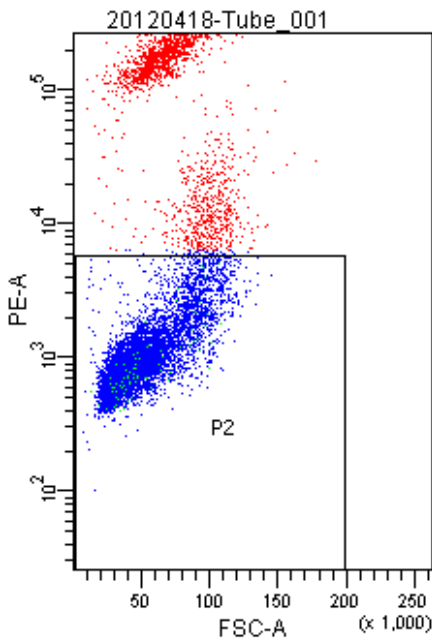

Tube: Tube\_001

| Population | #Events | %Parent | %Total |
|------------|---------|---------|--------|
| All Events | 10,000  | ####    | 100.0  |
| P1         | 9,887   | 98.9    | 98.9   |
| P2         | 8,018   | 81.1    | 80.2   |
| P3         | 80      | 1.0     | 0.8    |

Supplement: S1 File — (ZIP) [file pone.0152813.s001.zip › underlying images for plos one/Fig 3/Fig.3D-left panel-HeLa.pdf]

# FACSDiva Version 6.1.3

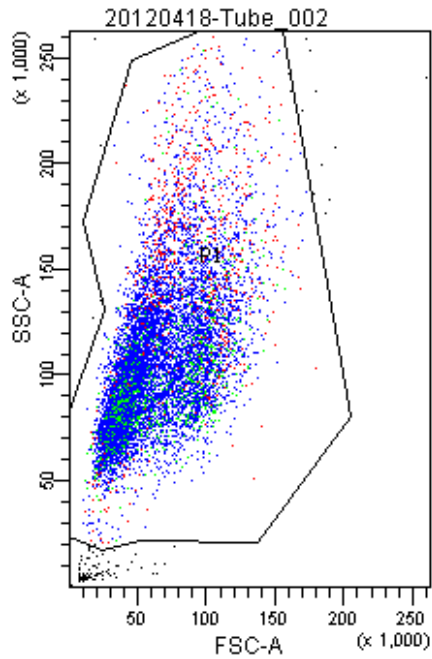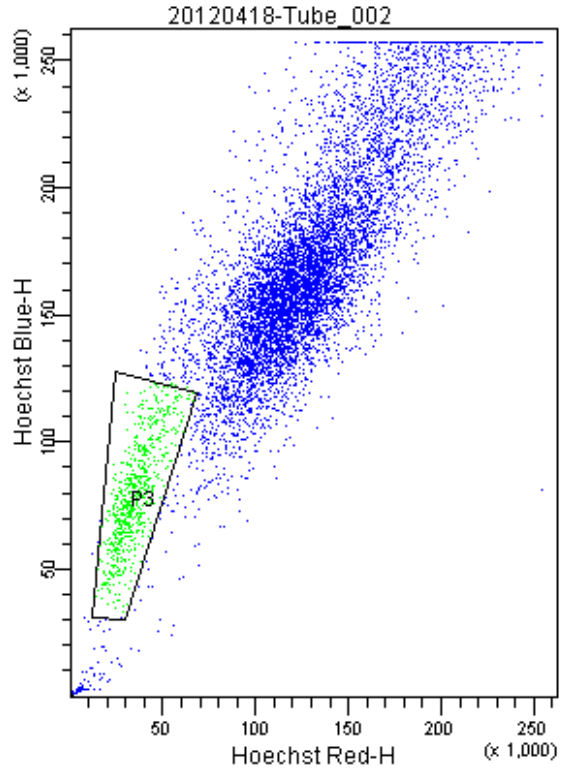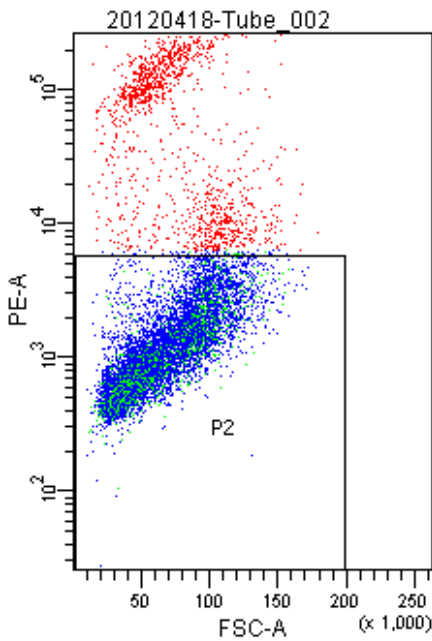

Tube: Tube\_002

| Population   | #Events | %Parent | %Total |
|--------------|---------|---------|--------|
| ■ All Events | 10,000  | ####    | 100.0  |
| ■ P1         | 9,806   | 98.1    | 98.1   |
| ■ P2         | 8,503   | 86.7    | 85.0   |
| ■ P3         | 705     | 8.3     | 7.0    |

Supplement: S1 File — (ZIP) [file pone.0152813.s001.zip › underlying images for plos one/Fig 3/Fig.3D-right panel-HeLa-TSA.pdf]

HeLa

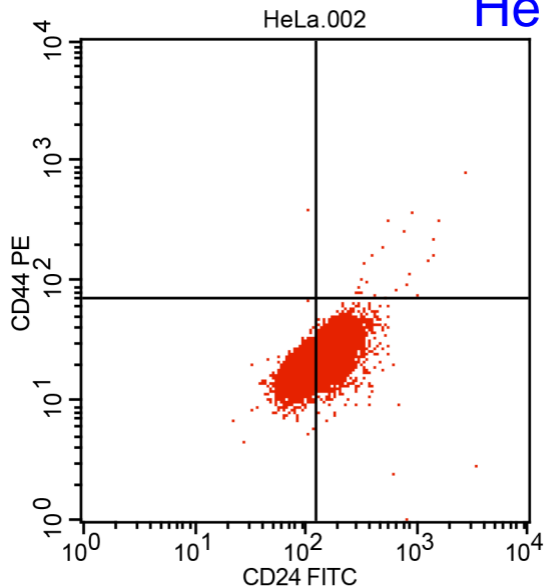

HeLa/TSA

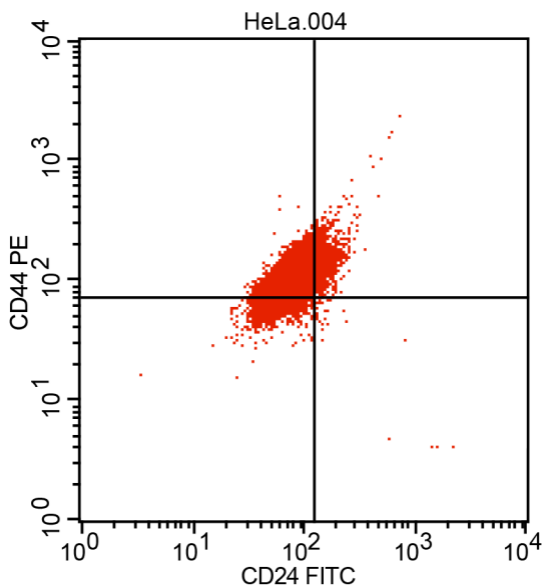

Supplement: S1 File — (ZIP) [file pone.0152813.s001.zip › underlying images for plos one/Fig 3/Fig.3E.pdf]

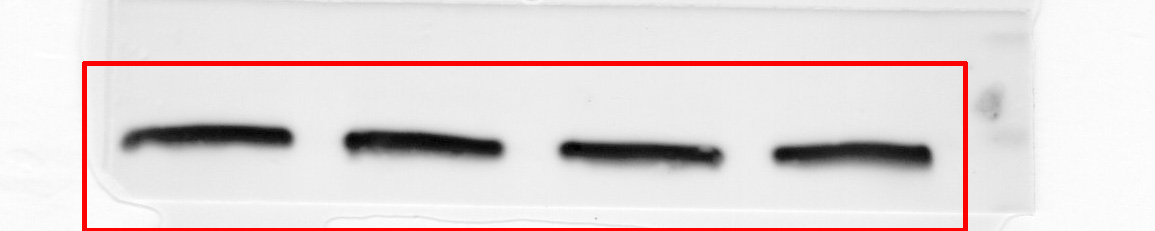

Supplement: S1 File — (ZIP) [file pone.0152813.s001.zip › underlying images for plos one/Fig 4/Fig.4B actin.jpg]

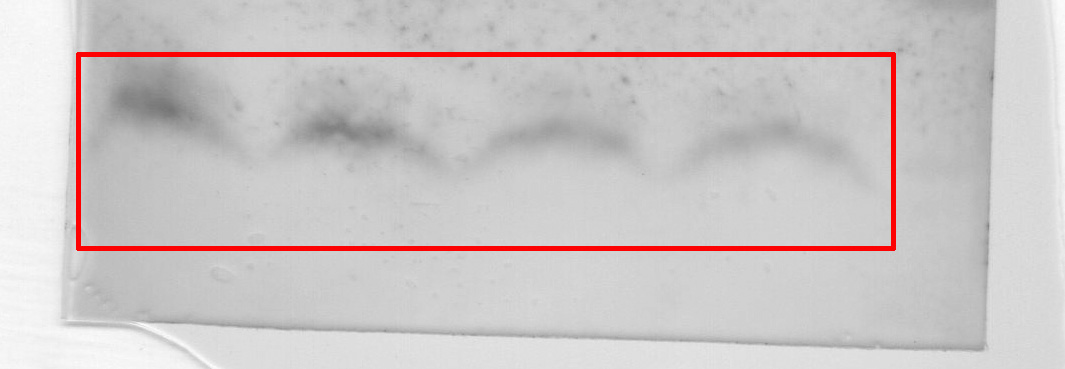

Supplement: S1 File — (ZIP) [file pone.0152813.s001.zip › underlying images for plos one/Fig 4/Fig.4B UbB.jpg]

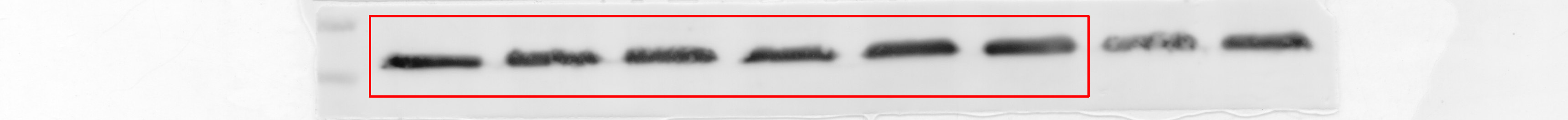

Supplement: S1 File — (ZIP) [file pone.0152813.s001.zip › underlying images for plos one/Fig 4/Fig.4C actin.jpg]

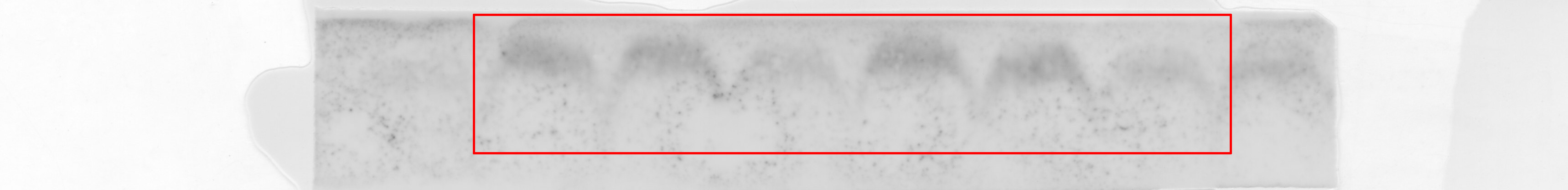

Supplement: S1 File — (ZIP) [file pone.0152813.s001.zip › underlying images for plos one/Fig 4/Fig.4C UbB.jpg]

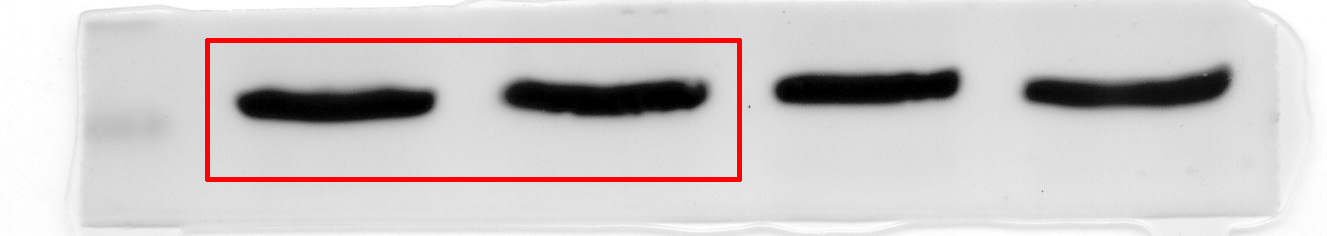

Supplement: S1 File — (ZIP) [file pone.0152813.s001.zip › underlying images for plos one/Fig 4/Fig.4F actin.jpg]

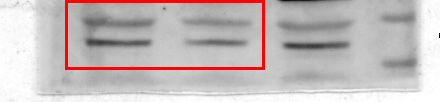

Supplement: S1 File — (ZIP) [file pone.0152813.s001.zip › underlying images for plos one/Fig 4/Fig.4F Nanog.jpg]

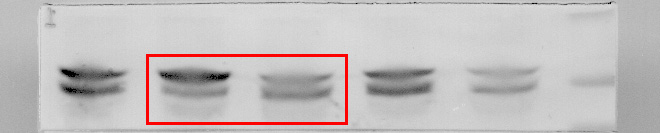

Supplement: S1 File — (ZIP) [file pone.0152813.s001.zip › underlying images for plos one/Fig 4/Fig.4F Oct4.jpg]

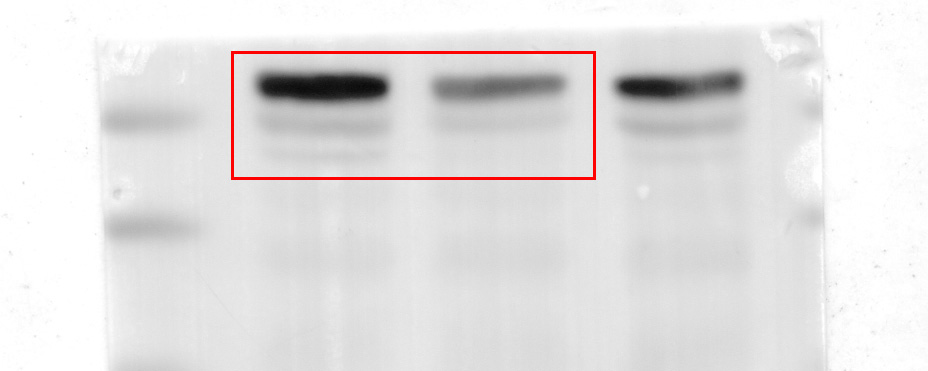

Supplement: S1 File — (ZIP) [file pone.0152813.s001.zip › underlying images for plos one/Fig 4/Fig.4F Sox2.jpg]

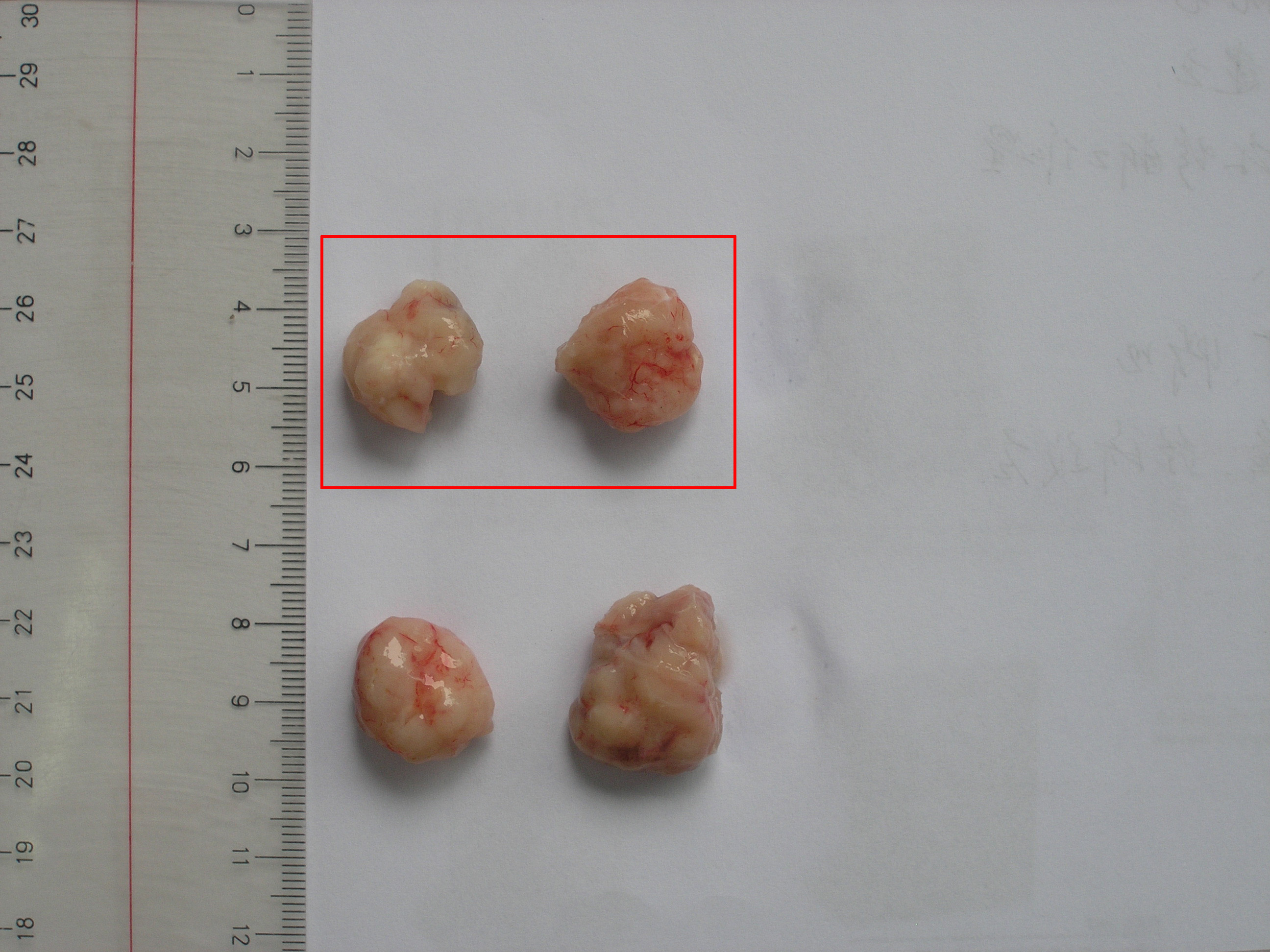

Supplement: S1 File — (ZIP) [file pone.0152813.s001.zip › underlying images for plos one/Fig 5/Fig.5A.jpg]

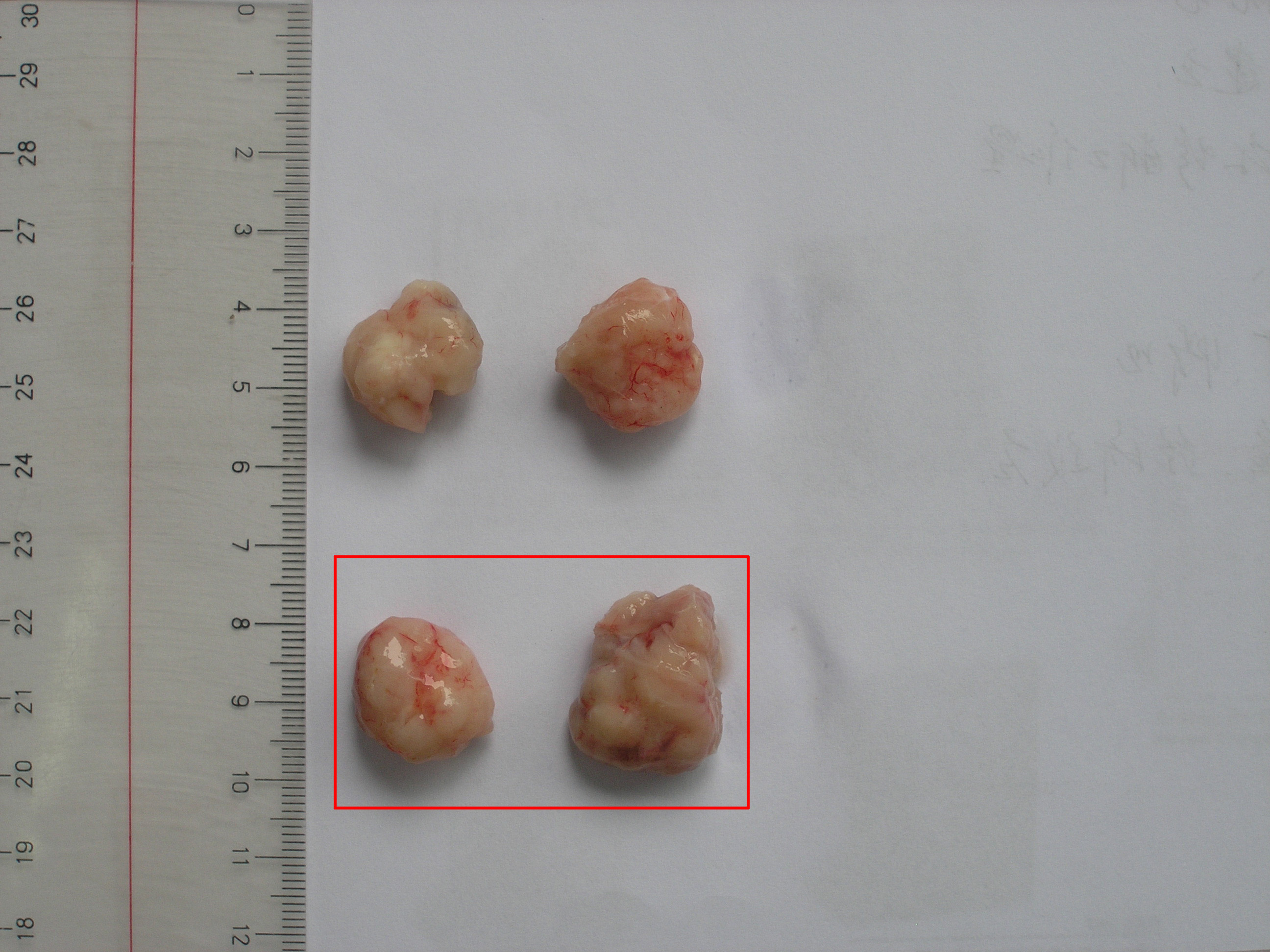

Supplement: S1 File — (ZIP) [file pone.0152813.s001.zip › underlying images for plos one/Fig 5/Fig.5B.jpg]

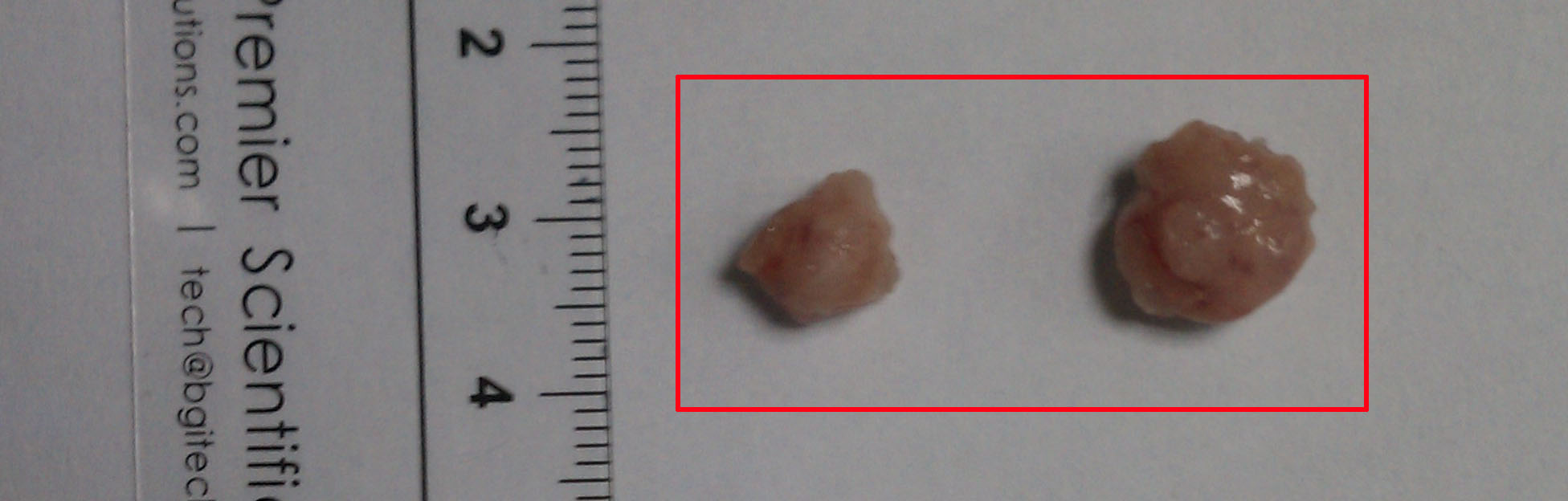

Supplement: S1 File — (ZIP) [file pone.0152813.s001.zip › underlying images for plos one/Fig 5/Fig.5C.jpg]

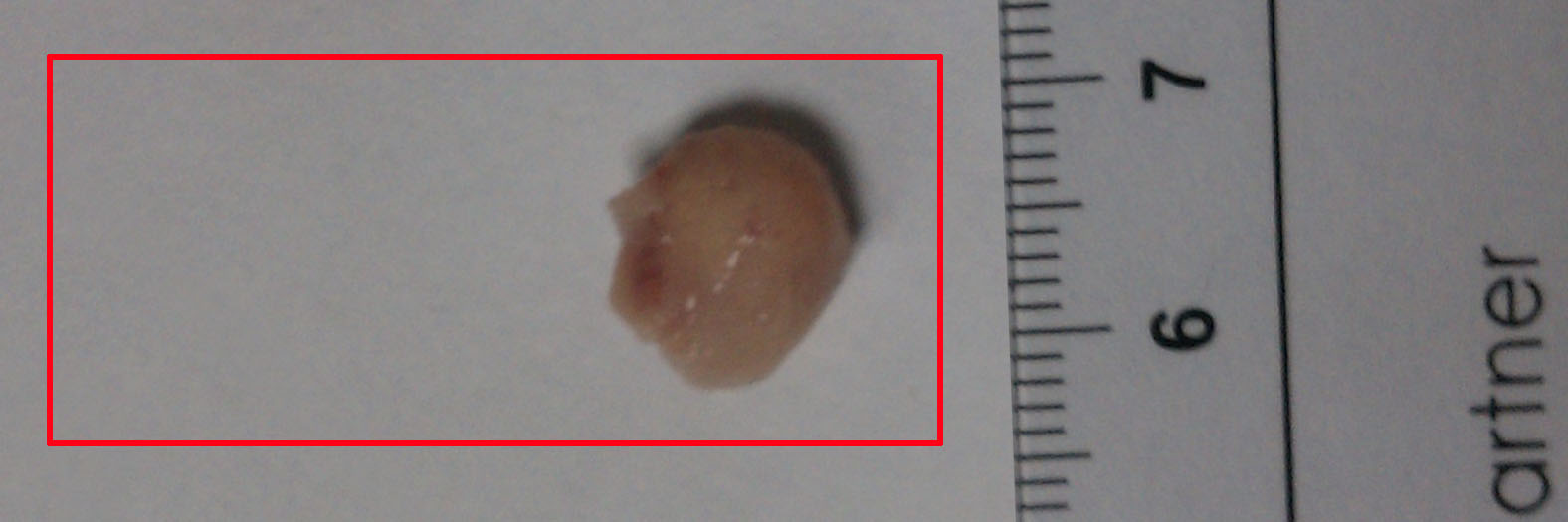

Supplement: S1 File — (ZIP) [file pone.0152813.s001.zip › underlying images for plos one/Fig 5/Fig.5D.jpg]

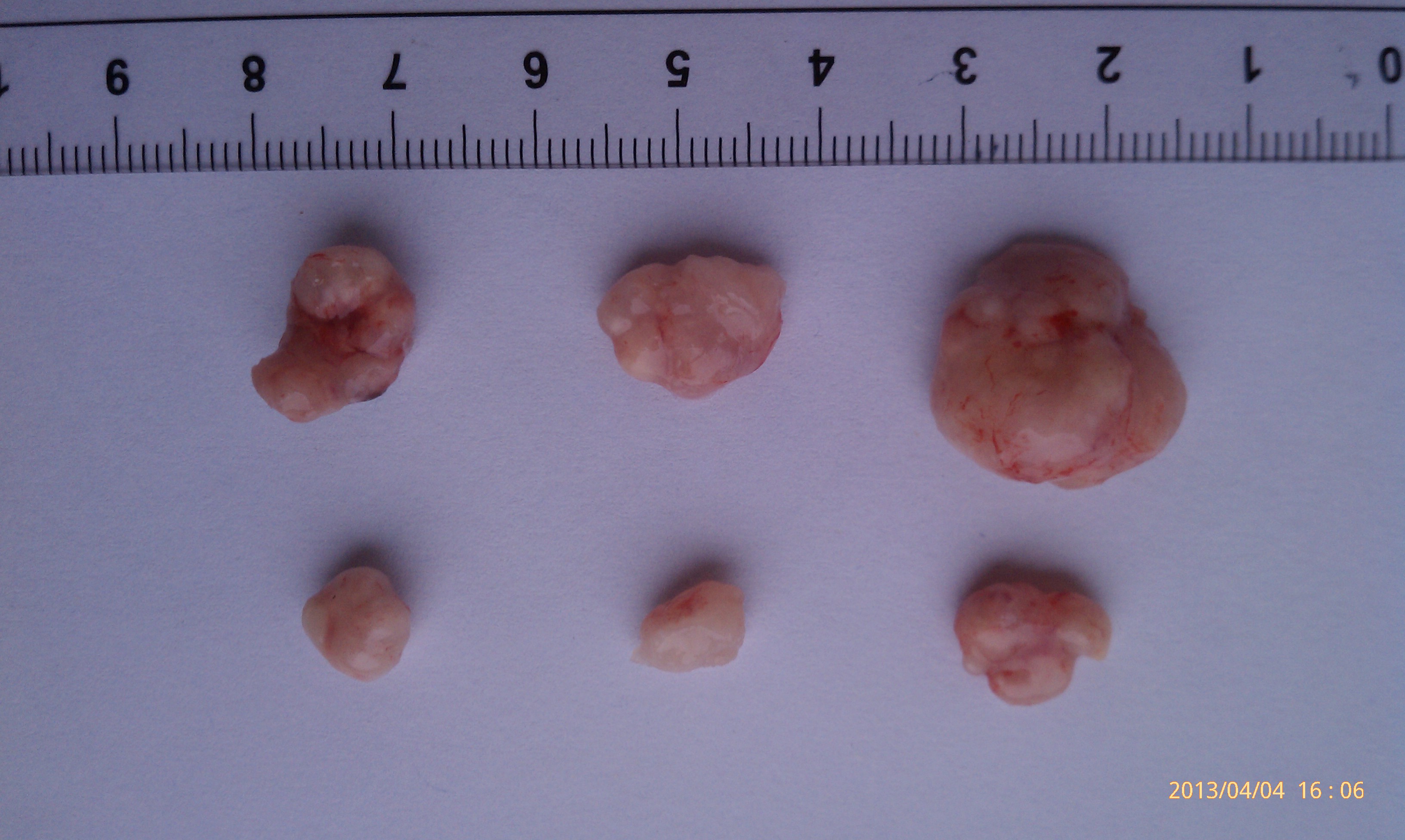

Supplement: S1 File — (ZIP) [file pone.0152813.s001.zip › underlying images for plos one/Fig 5/Fig.5F.jpg]
